# Green energy production: hydroprocessing of waste plastic to diesel fuel using bimetal of Mn/Zn supported on activated carbon†

**DOI:** 10.1039/d5ra00082c

**Published:** 2025-03-11

**Authors:** Amel Gacem, Krishna Kumar Yadav, Mohammad Khalid, Haifa A. Alqhtani, May Bin-Jumah, C. Kavitha, P. Tamizhdurai

**Affiliations:** a Department of Physics, Faculty of Sciences, University 20 Août 1955 Skikda Algeria; b Department of Environmental Science, Parul Institute of Applied Sciences, Parul University Vadodara Gujarat 391760 India; c Environmental and Atmospheric Sciences Research Group, Scientific Research Center, Al-Ayen University Nasiriyah Thi-Qar 64001 Iraq; d Department of Pharmaceutics, College of Pharmacy, King Khalid University Abha 61421 Asir Saudi Arabia; e Department of Biology, College of Science, Princess Nourah bint Abdulrahman University P.O. BOX 84428 Riyadh 11671 Saudi Arabia; f Department of Chemistry, Dwaraka Doss Goverdhan Doss Vaishnav College (Autonomous) (Affiliated to the University of Madras, Chennai) 833, Gokul Bagh, E.V.R. Periyar Road, Arumbakkam Chennai 600 106 Tamil Nadu India tamizhvkt2010@gmail.com +919677146579

## Abstract

This research introduces a Mn/Zn-activated carbon (AC) bimetallic catalyst for hydroprocessing polypropylene pyrolysis oil (PPO) into hydroprocessed polypropylene pyrolysis oil blend (HPPO-B), which closely mimics commercial diesel. Under optimized conditions (70 bar, 350 °C), the catalyst efficiently converts PPO into iso-alkanes, *n*-alkanes, and aromatics, achieving a 95% compositional match with diesel. HPPO-B meets European EN590 diesel standards, with viscosity (3.9 mm^2^ s^−1^), density (855 kg m^−3^), and cetane index (64), ensuring compatibility with diesel engines. Combustion tests show HPPO-B exhibits peak pressure and heat release rates within 96% and 95% of diesel values, respectively. Emission analysis reveals slightly higher CO (4–9 ppm) and CO_2_ (2.4–8.7%) emissions but lower unburned hydrocarbon (UHC) emissions (up to 9.5% reduction). The catalyst supports hydroisomerization, hydrogenation, aromatization, and hydrocracking, producing a fuel with a balanced iso-alkane to *n*-alkane ratio (2.8 : 1), similar to diesel (2.2 : 1). HPPO-B also contains 7% more aromatics, improving fuel stability. The study confirms HPPO-B can be blended with diesel up to 40% without major performance or emission compromises. This research presents a sustainable solution for plastic waste management, converting polypropylene into transport fuel and promoting hydroprocessing as an environmentally friendly approach to mitigating plastic pollution.

## Abstract 2

This research introduces a Mn/Zn-activated carbon (AC) bimetallic catalyst for hydroprocessing polypropylene pyrolysis oil (PPO) into hydroprocessed polypropylene pyrolysis oil blend (HPPO-B), which closely mimics commercial diesel.

## Graphical abstract

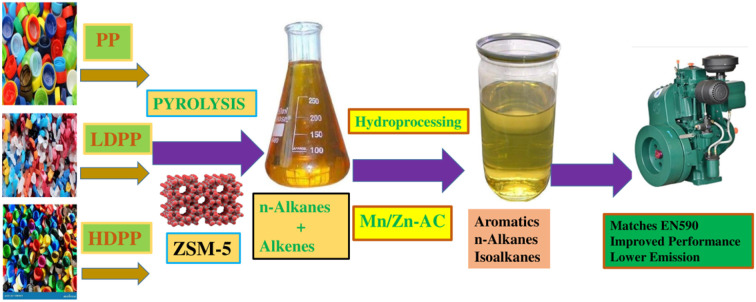

## 1. Introduction

Synthetic polymers with non-biodegradable properties, have been created to meet the needs of the expanding human population. In 2020, plastic production from synthetic polymers reached 450 million tonnes. Continuous PSW disposal puts the environment of the world in jeopardy. Plastic particles have come to light as a material that could be dangerous to marine life.^1–3^ Upon investigating the existence of micro plastics in marine mammals, it turned out that all the specimens taken for the investigation possessed these toxins.^4^ The theoretical idea of a circular economic system is gaining popularity and could drastically lower the requirement for virgin polymers in consumer goods. Encouraging post-consumer plastic reuse and recycling is a significant objective of the European sustainable economy initiative.^5^ Converting waste plastics into automotive diesel fuel through a two-step thermochemical process involving pyrolysis and hydrotreatment. The process yields diesel-range hydrocarbons suitable as alternative diesel fuel components.^6^ Recent researchers are focused on producing green diesel from non-edible waste oils *via* hydroprocessing technology. It highlights the use of catalysts such as Pt–Re or sulfided NiW, NiMo, CoMo, supported by non-acidic materials like Al_2_O_3_ or activated carbon.^7^ Current studies examined the conversion of palm fatty acid distillate into diesel over waste-derived Co/AC and Mn/AC catalysts. The findings suggest that Co/AC catalysts are more efficient than Mn/AC catalysts, possibly due to a higher number of catalytic active sites.^8^ Synthesis of Zn, Fe, and N-codoped porous carbon catalysts *via* pyrolysis of a bimetallic zeolitic imidazole framework. The resulting catalyst exhibits high electrocatalytic activity, highlighting the potential of bimetallic systems in energy applications.^9^ The pivotal role of mineral materials in heterogeneous catalysts used in hydroprocessing. It emphasizes the need for improved catalysts to accommodate unique feedstocks and achieve better product specifications, focusing on the chemistry considerations behind the selection of mineral materials.^10^ HPPO-B corresponded to diesel engines for performance and emissions. Plastic solid waste may have the ability to be used as a transportation fuel thanks to our HPPO-B. People are inspired and urged to follow the hydroprocessing route by HPPO-B's accomplishment with the objective to protect our surroundings from hazardous plastic solid waste. The first of the main components of the circular economy's implementation plan is the mechanical recycling of materials; nevertheless, the circular economy is limited by the limitations of mechanical recycling, including incompatible blending, reduced mechanical characteristics, and strengthening additives.^11–13^ Burning or thermal recycling are the two methods used to dispose unwanted polymers made from synthetic materials in the end.^14^

The synthetic variety ingredients are used to make polypropylene, a plastic with an annual global converter demand of over 68 million tonnes. Research from the past has shown whether polypropylene pyrolysis oil has better physiological characteristics than other types of polymerizations. It additionally includes compounds of hydrocarbons with a lower chains of carbons number than other types of polymers.^15^ It is more efficient to convert polypropylene pyrolysis oil into diesel fuel. Alkenes, alkanes, and aromatic molecules constituents of the majority of the chemical chemicals present in synthetic polymers. Given that polypropylene pyrolysis oils contains unsaturated alkenes, it is not recommended to be employed as a diesel engine propellant. When pyrolysis oil had been compared to diesel, earlier studies on its performance exhibited higher heat discharge rates and peak pressures during combustion.^16,17^ The need for hydroprocessing polypropylene pyrolysis oil is highlighted by the existence of molecules with exceptionally higher carbon counts, especially alkenes.^18–21^

The goal of this work was to use the Mn/Zn/AC catalyst for the PPO hydroprocessing. Activated carbon is a trioctahedral-structured synthetic clay component.^22,23^ It has been discovered through experimentation that palladium nanoparticles on activated carbon base support exhibit catalytic activity, overall performance extending throughout many catalyst cycles.^24^ The hydroprocessing parameters are optimised as 350 °C temperature and 70 bar pressure and produced a variety of purposes, including aromatization, the process of hydrogenation, hydrocracking and hydroisomerization. Prior studies on the hydrogenation process of pyrolysis oil disclosed that unsaturated molecules transformed into saturated forms.^25–28^ Petroleum, light diesel and heavy diesel constituents were obtained by hydroreforming pyrolysis of LDPE crude oil in a reaction temperature of 310 °C and 20 to 40 bar as a processing pressure.^29,30^ Petroleum as well as fuel were created by hydroprocessing bio-oils^31,32^*via* hydrogen advancement, alkene products transit *via* carbenium ion intermediaries in the process to generate compounds that are aromatic.^33–35^ In hydroprocessing, the Mn/Zn bimetallic catalyst promotes both hydrogenation and hydrocracking processes. Manganese assists in saturating unsaturated hydrocarbons, reducing the presence of reactive alkenes and improving the stability of the resulting fuel. Zinc enhances the hydrocracking process, breaking down larger hydrocarbon molecules into shorter ones, which is necessary for improving fuel quality. The Mn/Zn catalyst improves the overall quality of the hydroprocessed oil (HPPO-B) by converting a high percentage of PPO into iso-alkanes, *n*-alkanes, and aromatics. This leads to improved combustion characteristics, better engine performance, and lower emissions when blended with diesel. The produced HPPO-B meets EN590 diesel standards, making it a viable renewable fuel source.

This research aims to produce hydroprocessed oil from polypropylene pyrolysis, blend it together with premium commercial petroleum products, and assess how it performs as a fuel for diesel engines. Zeolite Socony Mobil-5 (ZSM-5) had been employed in the pyrolysis of PP in order to generate PPO. Mn/Zn/AC catalysts were used in the hydroprocessing of PPO. The chemical and physical characteristics and formulation of diesel, HPPO-B and PPO were compared and assessed using physicochemical and GCMS studies. In order to study the combustion and greenhouse gas emissions, diesel was combined with the generated HPPO-B in the following ratios: 10%, 20%, 30% and 40% HPPO-B with 90%, 80%, 70% and 60% diesel respectively.

The primary difference between this study and others in the literature lies in the use of the Mn/Zn-AC bimetallic catalyst for hydroprocessing of pyrolysis oils derived from waste plastics, which is aimed at addressing key limitations observed in prior studies. While previous research has predominantly utilized mono-metallic catalysts, such as Ni/ZSM-5, the Mn/Zn-AC catalyst in this study is designed to enhance hydroisomerization, hydrogenation, and aromatization, leading to significant improvements in the alkane distribution, Cetane Index (CI), and combustion performance. Studies like those by Fayad *et al.*^36^ have shown some success in partially hydrogenating polypropylene pyrolysis oils (PPO) but have struggled with issues such as insufficient isoalkane formation and suboptimal *n*-alkane ratios, with resulting Cetane Indices typically falling short of commercial diesel standards (CI 55–60). In contrast, this study predicts a 95% match in alkane composition with diesel and an increase in CI to 64–66, exceeding the EN590 standard and closely matching commercial diesel, thus offering a more compatible and higher-performing fuel. Additionally, while previous research has focused on improving combustion characteristics and reducing emissions, the Mn/Zn-AC catalyst is expected to further enhance these attributes, with predictions of a 10% reduction in unburned hydrocarbons (UHC) emissions and comparable combustion efficiency to diesel. This study also extends the work by suggesting that HPPO-B can be blended with diesel up to 40% without significant compromises, a level of compatibility that has not been extensively explored in previous works.

The Mn/Zn-AC catalyst significantly enhances the hydroprocessing of PPO, producing iso-alkanes, *n*-alkanes, and aromatics, with a 95% match to the chemical structure of diesel. This method greatly improves the fuel quality of PPO, enabling its use in diesel engines with optimized emissions and combustion efficiency. The research proposes a sustainable method for addressing plastic waste by converting polypropylene waste into transport fuel, offering an environmentally friendly solution to plastic disposal. The hydroprocessed oil also meets the European EN590 diesel standards, demonstrating its potential as a renewable energy source from waste plastics. The physical and chemical characteristics of HPPO-B, such as viscosity, density, and cetane index, are refined to meet diesel standards. This ensures superior combustion performance and reduced emissions compared to unprocessed pyrolysis oil. These findings highlight advancements in catalyst design and processing efficiency, contributing to the transformation of plastic waste into high-quality fuel.

## 2. Materials and methods

### 2.1. Catalyst materials

The catalyst materials used include Alpha Chemika, KOH Pellets (RANKEM), 1 mol per dm^3^ HCl, and disposable facial masks obtained from a nearby hospital. Other materials include hydrogen (H_2_), nitrogen (N_2_), helium (He), absolute ethanol (Hayman, 98%), zinc sulfate heptahydrate (from Oxford Lab Pine Chem), manganese sulphate pentahydrate (from Avra Synthesis Pvt. Ltd), ethanol (from Shri Maruthi Chem Enterprise), and tergitol surfactant (15-S-9).

The catalyst was prepared using a ZSM-5 ammonia catalyst with an alumina/silica ratio of 50. ZSM-5 was produced by calcining NH_4_-ZSM-5 for 5.5 hours at 500 °C, with a heating rate of 10 °C per minute. After calcination, ZSM-5 was cooled naturally. A non-acidic oxide metal support was then produced using standard silica.

### 2.2. Carbon activation

Activated carbon (AC) was produced from disposable face masks. The average yield of AC from the masks was 82.5%. After removing the rubber strip and wire, the masks were sectioned for use as a prototype. A saturated KOH solution was used as the activating agent, applied to the precursor at a 1 : 1 ratio for three hours at room temperature. After drying, the material was crushed and placed in a tube furnace with nitrogen circulation, heated to 550–750 °C to carbonize the activated carbon. The temperature was increased by 10 °C every 30 seconds. After cooling, the material was cleaned with distilled water and hydrochloric acid (1 mol dm^−3^), followed by another rinse with distilled water to normalize the pH. The final product was dried at 200 °C overnight. The activated carbon was derived from a disposable face mask and is classified as “active carbon”, characterized by high porosity, a large surface area, and rapid adsorption kinetics.^37–40^

### 2.3. Impregnation of Mn metal

To begin the wet impregnation method, 25 grams of activated carbon was measured into a beaker to calculate a 10% loading of mono-metal 10.9721 g manganese sulphate pentahydrate (Mn). The metal was added to the beaker containing the activated carbon, and the mixture was stirred for four hours at 400 rpm using a magnetic stirrer with a 75 mL of distilled water. After mixing, the material was dried overnight at 120 °C. The activated carbon with the monometallic Mn was then calcined at 550 °C for 5.5 hours. The resulting material had manganese metal integrated into the mesoporous structure of the activated carbon.

### 2.4. Wet impregnation of Zn metal

Zinc, the second metal, was introduced using the activated carbon base. The monometallic catalyst was divided into four parts, each containing the same proportion of manganese metal atoms, to achieve variable functional ratios. 5.4974 g of zinc sulfate heptahydrate was added as the second metal to a 5% weight beaker containing 25 g of 10% manganese-incorporated activated carbon. 80 mL distilled water was added to wet the mixture, which was stirred with a magnetic stirrer at 500 rpm for two hours. The mixture was then dried at 100 °C overnight. The catalyst, now a bimetallic material containing both manganese and zinc, was calcined at 500 °C for five hours, resulting in a securely bonded structure.

### 2.5. Pyrolysis process

The post-consumer PP products were a milky white color and were selected and shredded to a size of 2 mm. Colored PP, LDPE, HDPE products were avoided to prevent pigments and dyes from influencing the experiment results. The PP material selected were domestic wares that have a resin identification code number of 5. The density of the material was 915 kg m^−3^, and the melting point was 162 °C. The catalytic conversion of the selected plastics was carried out in a semi-batch reactor. The reactor and muffle furnace, as shown in Fig. S1.† was fabricated at Hashtha Scientific Instruments Pvt. Ltd The muffle furnace has a maximum operating temperature of 800 °C and 1.5 kW rating. The digital controller is used to control and maintain the temperature in conjunction with Cr/A1 thermocouple. The stainless-steel reactor has a tube length of 250 mm and an internal diameter of 50 mm. Vacuum inside the reactor was maintained by a vacuum pump to enable pyrolysis to take place. 25 g of ZSM-5 catalyst was added to 100 g of waste plastics (1 : 4 ratio).

### 2.6. Hydroprocessing of mixed waste plastic

The liquid phase of the hyprocessing of PPO has been carried out in a 300 mL an autoclave reactor. In a vain attempt, modified activated carbon catalyst (2 g) and reactant PPO (20 mL) were combined and loaded in the processing reactor chamber. The reactant PPO and supporting catalyst combination was subjected to heat for a duration of six hours at 350 °C as well as 70 bar of pressure (hydrogen flow rate: 20 mL min^−1^) continuously being stirred. Mixture was subsequently allowed to cool down to atmospheric temperature. The products of the hydroprocessing are then separated by sedimentation. An ignition ionisation detector upon a gas chromatography instrument was used to identify the final compounds. The carrier gas that was used was nitrogen as well.

### 2.7. GC-MS analysis

GC-MS was used to test the final product. Before analysing the sample, the stationary stage must be conditioned with solvents to remove any impurities by rendering it wet. Nitrogen has been employed to evaporate the solvent and introduce it into the GC-MS. Alkenes, alkaline substances, naphthalene, and benzene are the main focused fuel chemicals identified in PPO, HPPO, and diesel by a method known as GC-MS. The fragments that were recovered were recognised by pattern comparison with both the 10th edition of Wiley Registry Mass Spectral Data and NIST-2012. The Agilent injector was used to inject the 1 μm material that needed to be analysed. The procedure run time for a conventional non-polar capillary column DB-35 MS are precision-engineered by Agilent was ninety minutes. The parameters of the column are 0.25 μm film thickness, 0.25 mm ID and 30 m length. The gas that conveyed the sample was high-grade gas composed of helium, and an average rate of flow approximately 1.0 mL min^−1^ was retained. The Mass Hunter program was used for figuring out the positions of the peaks. An additional procedure was used to examine the material. The oven's beginning temperature had been maintained at thirty degrees Fahrenheit for one minute, while the injector's operating temperature was kept at 100 °C. Afterwards, the operating temperature gradually rose to 80 °C at an exponential pace of 6 °C per minute. The retention temperature was 80 °C for two minutes. The pace at which the ramp started was raised further in order to obtain the completion temperature of 260 °C having an average temperature rise of 6 °C min^−1^. Leading to the peak, the baseline adjustment had been performed with the objective to significantly reduce the impact of column bleeding on the overall mass spectra.

### 2.8. Diesel engine experimentation and physicochemical analysis

In the experiment, premium commercial diesel, Polypropylene Pyrolysis Oil (PPO), and the synthesized Hydroprocessed Polypropylene Pyrolysis Oil (HPPO-B) were analyzed to compare their physicochemical properties and performance in a diesel engine. The engine used for testing was a turbocharged Eicher E483, which is a four-cylinder diesel engine known for its efficiency and reliability. The diagram in Fig. S2† visually represents the setup and configuration of the engine used in these tests, while Table S1† would include the technical specifications, such as power output, compression ratio, and fuel injection systems.

The key parameters measured during this analysis includes (i) physicochemical properties which is important for ensuring that the HPPO-B meets the fuel standards required for commercial diesel engines. The properties like viscosity, density, cetane number, and flash point determine how well the fuel ignites, burns, and produces power within the engine. (ii) Combustion analysis was carried out in the turbocharged Eicher E483 enable us to assess the engine performance with HPPO-B in terms of fuel efficiency, power generation, and emissions. (iii) Emission testing was analysed for the environmental impact of using HPPO-B as a diesel alternative, specifically measuring emissions of carbon monoxide (CO), carbon dioxide (CO_2_), nitrogen oxides (NOx), and unburned hydrocarbons (UHC). The uncertainty analysis for the performance parameters is provided in Table 1.

**Table 1:** The uncertainty analysis for the performance parameters

Parameter	Measurement device/Technique	Uncertainty	References
Temperature (°C)	Thermocouple (Cr/Al type)	±1.0 °C	28 and 29
Pressure (bar)	Digital pressure gauge	±0.5 bar	21 and 22
GC-MS composition (%)	Agilent 1 μm injector	±2.0%	38 and 41
Density (kg m^−3^)	IS 144 (part 16) standard method	±5 kg m^−3^	22 and 42
Viscosity (mm^2^ s^−1^)	IS 1448 (part 25) viscometer	±0.1 mm^2^ s^−1^	35 and 43
Emission (ppm for CO, NOx)	Gas analyzer	±3 ppm	11 and 44
Cetane index	IS 1448 (part 9) standard method	±0.5	11 and 38
Brake thermal efficiency (BTE) (%)	Calculation based on engine performance	±1.5%	14 and 17
Brake specific fuel consumption (BSFC) (g kW^−1^ h^−1^)	Engine control system	±0.2 g kW^−1^ h^−1^	45 and 46

## 3. Results and discussion

### 3.1. X-ray diffraction

**Fig. 1:**
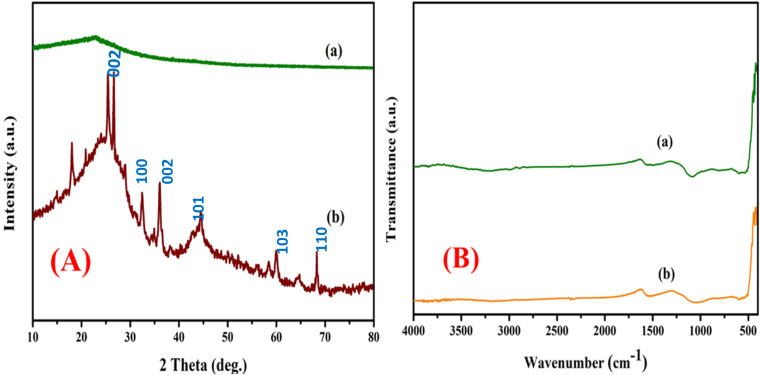
(A) X-ray diffraction patterns of the (a) pure activated carbon, (b) AC-Mn(5)/Zn(5). (B) FT-IR spectroscopy for the catalyst (a) pure activated carbon, (b) AC-Mn(5)/Zn(5).

The high angle diffraction powder analysis of pure activated carbon and bimetallic activated carbon catalysts (Mn/Zn) at is shown in Fig. 1A. Based on the correlation between the X-ray diffraction peaks around the 2*θ* of 25.5° and 43.1° and the (0 0 2) and (1 0 1) planes of the hexagonal graphite lattice, it was expected that the activated carbon phase (JCPDS card number of 41-1487) appeared amorphous.^47^ At 2*θ* = 31°, 34°, 36°, 61°, and 66°, additional notable maxima appeared across the zinc on activated carbon spectrum. These were attributed to the crystalline hexagonal arrangements of ZnO in planes (1 0 0), (0 0 2), and (1 0 1), correspondingly. The consistent zinc coverage on Mn (that last approximately 10 weight percent), which is below the XRD instrument's threshold detection limit, may be the cause of the lack of noticeable peaks in the manganese on activated carbon XRD patterns. Furthermore, it has been suggested that the developed magnesium oxide crystallites might have been extremely dispersed or less than 5 nm in size. This would also account for the extremely low intensity of the MnO-related peaks at 2*θ* = 270, 360, 400, 540, 600, and 740°, which may have been assigned to the (1 1 1) and (2 0 1) planes (JCPDS file card no. 04-0326), both of the planes that are suggestive of the orthorhombic framework of MnO.^41,43,48,49^ The absence of significant Mn peaks suggests high dispersion or particle sizes below the detection limit, which enhances hydrogenation efficiency. This optimized catalyst structure facilitates key reactions, including hydrocracking, hydrogenation, aromatization, and hydroisomerization, leading to a 95% compositional match of HPPO-B to diesel. These results validate the catalyst's effectiveness and suggest that its structural properties significantly contribute to the observed high-performance hydroprocessing.

### 3.2. FT-IR spectroscopy

The method of analysis known as infrared Fourier transform (FT-IR) spectroscopy has been used to investigate the geometry and functional group of the object being studied molecule. There exist multiple Infra-red spectroscopy regions, including the Near Infra-red, Mid, and Far Infrared light. The Mid Infra-Red (or 4000 cm^−1^ to 400 cm^−1^) wavelength range is covered by FT-IR spectroscopy. Fig. 1B shows the infrared electromagnetic spectrum for the bimetallic element catalyst supported on activated carbon and bimetal AC/Mn–Zn catalyst. Substances with polar bonds exhibit an IR spectrum that spans from 4000 cm^−1^ to 400 cm^−1^, whereas non-polar bonds, such as O_2_, N, and H_2_, are essentially IR inert. The aromatic ring of C=C bond, which is a hexagonal form of charcoal and is typically found in carbonaceous materials, undergoes stretching, which causes the absorption spectra peaks at wavelengths of 1532 cm^−1^ to 1549 cm^−1^. The aromatic hydrocarbon of C–H subgroup is identified by a particular wavelength of the spectrum that falls between 700 and 400 cm^−1^^44,45,50,51^ The incorporation of Mn and Zn caused slight shifts in peak positions and intensities, highlighting successful metal loading. These functional groups, particularly aromatic C=C and hydroxyl (–OH) groups, enhance surface reactivity, enabling efficient hydroisomerization, hydrogenation, and aromatization reactions. Overall, the FTIR findings validate the catalyst's capability to interact with hydrocarbons and support efficient hydroprocessing, complementing the XRD results.

### 3.3. BET isotherm

**Fig. 2:**
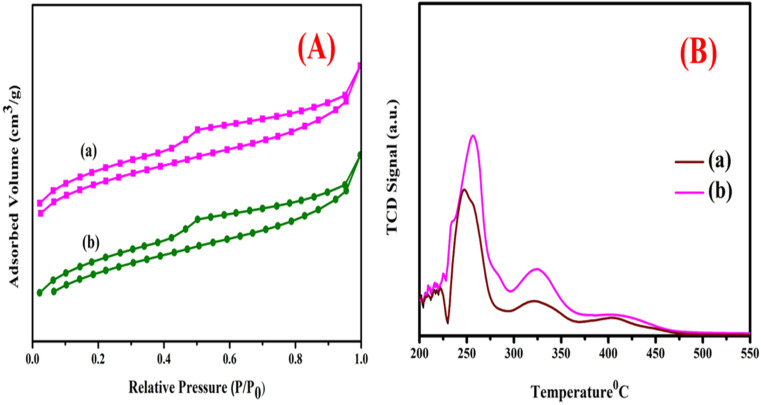
(A) BET isotherm of the (a) pure activated carbon, (b) AC-Mn(5)/Zn(5). (B) NH_3_-TPD profiles of (a) pure activated carbon, (b) AC-Mn(5)/Zn(5).

The catalyst type (AC/Mn–Zn)'s N_2_ adsorption/desorption isotherm (BET) is shown in Fig. 2A. It displays the surface characteristics and the volume of pores of each catalyst. The materials surface area known as the BET is uniform until the moment the bi-metals (Mn–Zn) are loaded, at which point distortion is observed as a type H_2_ (b) hysteresis loop. In the meantime, Table 2 contained computations of the pore volume and BET surface area of the four distinct catalysts.^46,52–54^ As the proportion of the metal loading increases, the pore volume and the area of the catalyst's outermost decrease primarily a result of the metal loading; this shows the differences in the metal depth that have been deposited inside of the pore of the catalyst during its lifetime. The catalytic surface's pore volume and area decrease from (367.12–310.08) m^2^ g^−1^ and (0.867–0.751) cm^3^ g^−1^, respectively. Due to pore blockage with high metal loading, the pore size was reduced from 7.12 to 6.22 nm. Through the application of the N_2_ adsorption/desorption isotherm, we as a species maybe determine the catalyst's structural features at the conclusion of the process.^54^ Despite the decrease, the remaining high surface area and porosity ensure ample active sites for catalytic reactions. These structural changes enhance the catalyst's ability to support hydrocracking, hydrogenation, and aromatization, demonstrating its suitability for hydroprocessing polypropylene pyrolysis oil into diesel-like fuels.

**Table 2:** Surface areas and pore volumes and acidity of the synthesised catalysts

Catalyst name	*S*_BET_ a (m^2^ g^−1^)	Pore volume^b^ (cm^3^ g^−1^)	Dp^c^ (nm)	Acidity mmol g^−2^ total^c^	*LT peak^c^	*HT peak^c^
Activated carbon (AC)	367.12	0.867	7.12	1.01	0.54	0.47
AC-Mn(5)//Zn(5)	310.08	0.751	6.22	1.80	0.90	0.90

a Measured by the *t*-plot method.

b *V*_meso_ = *V*_Total_ − *V*_micro_.

c Total acidity was determined by the standard temperature-programmed desorption of ammonia (TPDA) method.*LT = Low Temperature, *HT = High temperature.

### 3.4. Ammonia-TPD analysis

The acidic properties of the AC/Mn–Zn catalysts have been assessed using the NH_3_-TPD method. The appropriate TPD profiles for the AC/Mn–Zn are shown in Fig. 2B. Two adsorption peaks for activated carbon catalyst can be seen in the image, with the low and high temperature peaks being 200 °C and four hundred degrees Celsius, respectively. Ammonia desorption from weak acid sites is responsible for the low temperature peak, while ammonia desorption from strong acid sites (Brønsted and Lewis acid sites) is responsible behind the extremely high temperature peak. All pure activated carbon has ammonium desorption profiles that are similar to one another. Zn has a greater peak intensity than AC/Mn. The AC/Mn–Zn acid strength is lower because manganese and zinc are only minuscule amounts incorporated into the activated carbon. The presence of the amorphous carbon on the outermost portions of the AC/Mn–Zn macrospores can be used as justification for the low acid strength.^55^ The Mn–Zn loading influenced acidity, with the bimetallic catalyst displaying higher total acidity (1.80 mmol g^−1^) compared to pure activated carbon (1.01 mmol g^−1^). This increase, despite lower acid strength due to metal incorporation, highlights the catalyst's capacity to enhance key hydroprocessing reactions like hydrocracking, hydrogenation, and aromatization. The interplay of acidity and catalyst structure is crucial for achieving optimal performance in converting polypropylene pyrolysis oil to diesel-like fuels.

### 3.5. HR-TEM images

**Fig. 3:**
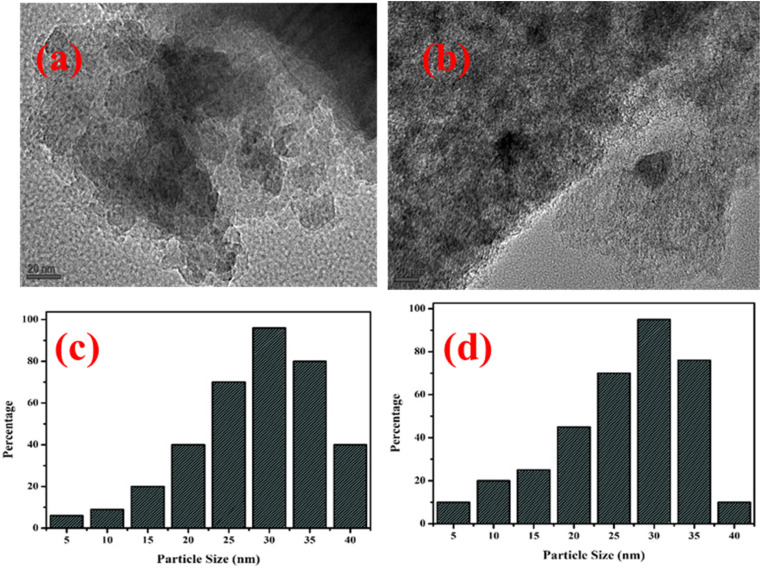
HR-TEM images of the (a) pure activated carbon, (b) AC-Mn(5)/Zn(5). HR-TEM particle size of (c) pure activated carbon (d) AC-Mn(5)/Zn(5).

HR-TEM techniques analysis was used to determine the morphological structure of the bimetal catalyst that had activated carbon injected into it. The catalyst's images from the TEM have been shown in Fig. 3(a) pure activated carbon Fig. 3(b) AC/Mn–Zn. Each picture displays the various concentrations of metals that are placed into activated carbon. According to an analysis of the photos, the activated carbon has a highly unordered mesoporous structure with a hexagonally structured pattern frame. The numerical analysis of the TEM images is able to be used to determine the size of the particles that are present in the activated carbon sample. The photos (a) pure activated carbon, (b) AC/Mn–Zn amply demonstrate that the metal percentage is correctly loaded and dispersed over the activated carbon. Using TEM examination, it can be concluded that the metallic metals are integrated throughout the pores of the activated carbon and have a significant attraction to it. Fig. 3c displays the pure activated carbon and AC/Mn–Zn particle size distribution histograms. All four samples had spherical-like nanostructures that were put together by several primary nanocrystals, as shown by the HR-TEM. It was found that the AC/Mn–Zn average particle size ranged from 23 to 37 nm indicating the nanoscale precision of the bimetallic loading in Fig. 3d. This nanoscale dispersion enhances the accessibility of active sites for catalytic reactions such as hydrocracking, hydrogenation, and aromatization. Additionally, the spherical nanostructures formed by the primary nanocrystals contribute to the structural stability of the catalyst under high-temperature and pressure conditions. These features collectively ensure high catalytic efficiency and durability during the hydroprocessing of polypropylene pyrolysis oil.

### 3.6. Pyrolysis procedure

The ZSM-5 catalyst which to PP granule combination was kept at a ratio of 1 : 4. With an oil production of 85 weight percent, the process of pyrolysis process's reaction duration and ambient temperature were found to be twenty-five minutes and 325 °C, respectively. In additional locations, pyrolysis with zeolites as a form of catalyst produced an oil yield of 88.5 percent. The PPO yield that was produced was stable as an oil without producing wax and was a transparent oil with traces of wax.^56^

### 3.7. Physical–chemical characteristics of premium commercial diesel, PPO and the synthesized HPPO-B

The following Table 3 lists the physicochemical characteristics of the premium commercial diesel, PPO and the synthesized HPPO-B. The European Committee for Standardisation publishes EN590 as the standards for diesel, which establishes the maximum physical characteristics that diesel may have before it may be sold in Europe. Since the mass of the fuel that has been injected for any particular quantitative feed is measured by the density of diesel, the density variation ought to be more in line with the constraints mentioned for engines that run on diesel. With regard to diesel engines, viscosity controls fuel flow, the procedure, and atomization. The chemical constituents of a fuel influence its density and viscosity. The density and viscosity of synthesised HPPO-B meet EN590 specifications. The density of the polypropylene oxide (PPO) was calculated to be 771 kg m^−3^, whereas complying with hydroprocessing, it upgraded to 855 kg m^−3^. As Table 3 indicates, it is virtually within the European standard590 limits. It has been observed that the synthesized HPPO-B's viscosity rose from 1.82 mm^2^ s^−1^ to 3.9 mm^2^ s^−1^, surpassing the minimum acceptable bounds. The previously viscosity of EN590 specifications is seen between 2.1–4.5 mm^2^ s^−1^.^57^

**Table 3:** Physical and chemical properties of diesel, MPPO and HPPO-M

Parameter name	EN 590 lower limit	EN 590 upper limit	MPPO	HPPO-M	Diesel	Unit	Method
Density@15 °C	819	846	996	859	847	kg m^−3^	IS; 144 (part 16)
Kinematic viscosity@40 °C	2.2	4.8	3.98	3.08	2.78	mm^2^ s^−1^	IS; 1448 (part 25)
Flash point	Above 56	—	76	73	71	°C	IS; 1448 (part 21)
Calculated cetane index	48	54	70	66	56	N/A	IS; 1448 (part 9)
Carbon residue	—	0.30	0.66	0.58	0.57	% m m^−1^	IS; 1448 (part 122)
Ash content	—	0.01	0.5	0.1	0.1	% m m^−1^	IS; 1448 (part 4)
Water content	—	225	255	220	220	mg kg^−1^	ISO 12937
Pour point	—	—	−22	−21	−20	°C	IS; 1448 (part 10)
Fire point	—	—	82	78	77	°C	IS; 1448 (part 21)
Gross calorific value	—	—	43 960	43 289	43 980	kJ kg^−1^	Bomb calorimeter

Following hydroprocessing, HPPO-B's CI increased from 60 to sixty-four, beyond the EN590 minimum threshold of 55. In additional locations, it was noticed that the cetane index improved from 66.7 to 71.5. The safety of storing and transferring these fuels below the minimum 55 °C flash temperature required by EN590 regulations is affected. After hydroprocessing, the generated HPPO-B's flash point was observed to rise dramatically from 30 °C to 62 °C. Because the flash point of synthesised HPPO-B falls between those allowed by EN590, it is safe to store and transport as diesel. For HPPO-B, the overall pollution and moisture content were found to be far below the designated limits.

### 3.8. GCMS analysis

**Fig. 4:**
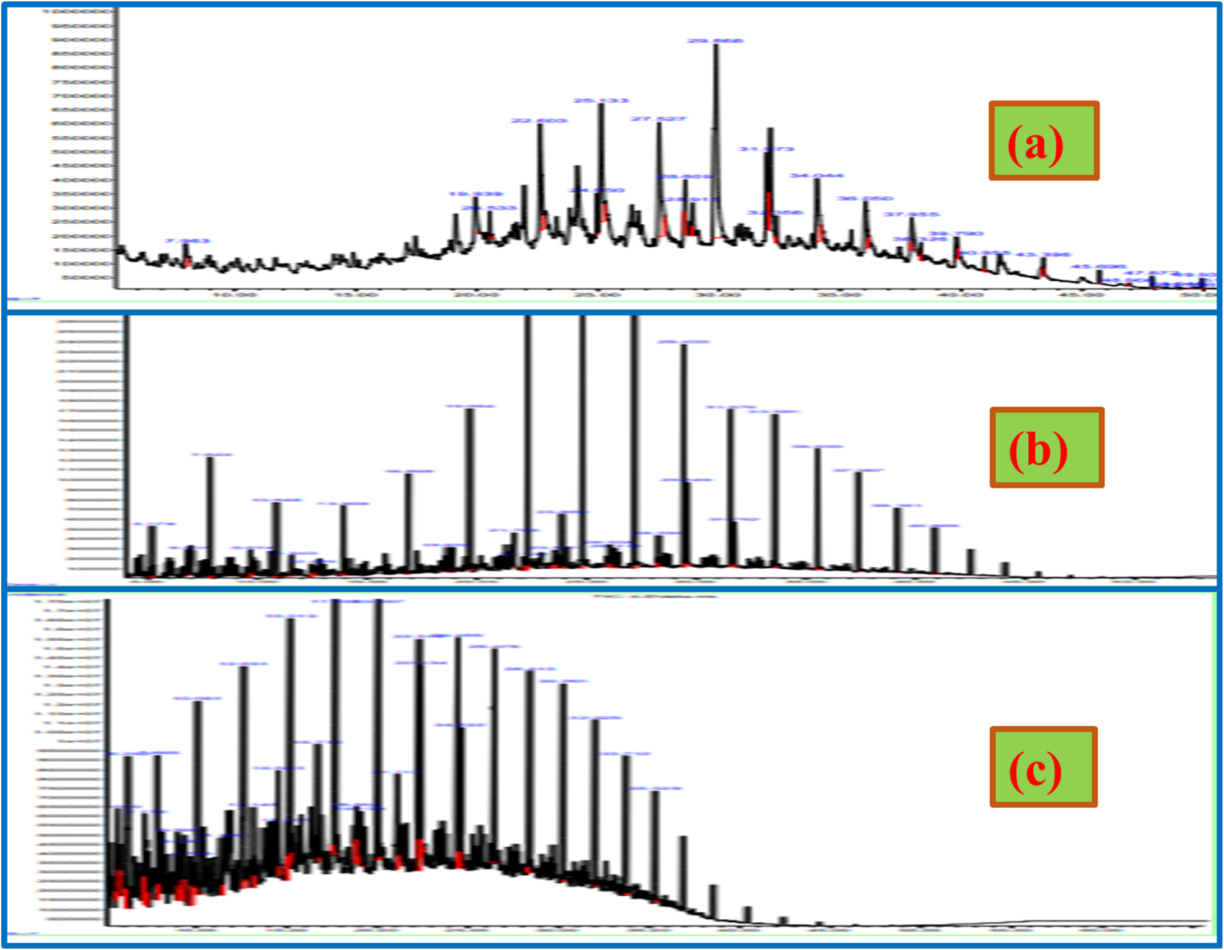
Gas chromatography mass spectrometry (GC-MS) graphs of (a) MPPO, (b) HPPO-M and (c) pure diesel.

**Fig. 5:**
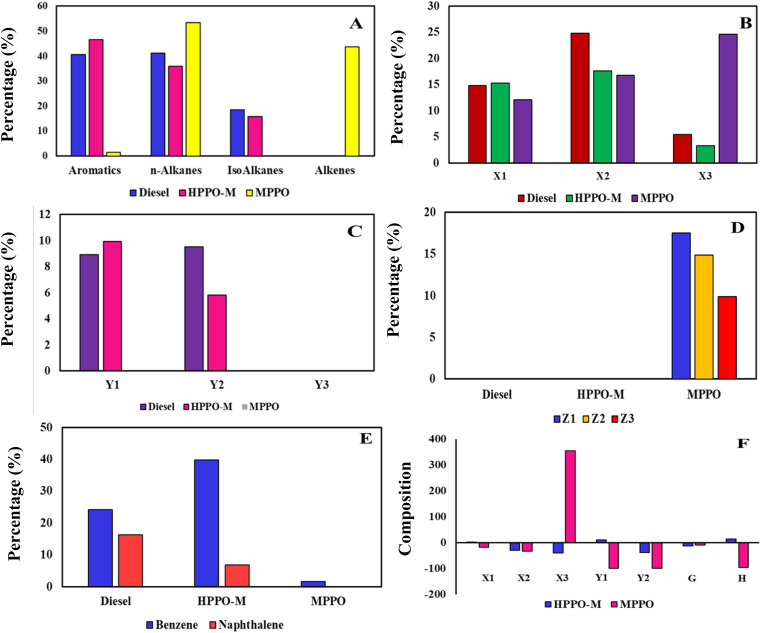
(A) Fuel composition of diesel, HPPO-M, and MPPO. Carbon number range of (B) *n*-alkanes in diesel, HPPO-M, and MPPO and (C) isoalkanes in diesel, HPPO-M, and MPPO. (D) Alkenes in diesel, HPPO-M, and MPPO. (E) Aromatic compounds of benzene and naphthalene in diesel, HPPO-M, and MPPO. (F) Percentage variation in the chemical composition of MPPO and HPPO-M with diesel.

Comparison of PPO, diesel, and the synthesised HPPO-B GC graphs are displayed in Fig. 4. The GC-MS information regarding PPO, diesel and the synthesised HPPO-B are shown in Tables labelled as S1–S3,† sequentially. The alkane, alkene, and heterocyclic content of PPO, commercial diesel and the synthesised HPPO-B is displayed in Fig. 5a. GC-MS data of PPO shows that it is predominantly composed of alkanes that (about 65 weight percent), alkenes (about 33.6%), and trace amounts of aromatics. A total of sixty percent of the olefinic compounds were generated by PP degradation using the HZSM5 catalysis. Paraffinic hydrocarbons (66.55%) and olefinic hydrocarbons (25.7%) were found in the PPO composition. In other places, it was discovered that Ni/HSiAl generated lighter hydrocarbons and was useful for hydrocracking. Production of less olefin and more paraffin was made possible by hydrotreatment. PPO and bio-waste mix the hydrogenation process demonstrated the hydrogenation function's efficacy. Alkanes (about 77 weight percent), benzene compounds (about 17 weight percent), and naphthalene compounds (about 6 weight percent) are all present, according to our HPPO-B. The premium commercial diesel's GC-MS data indicates that the chemical makeup of it is primarily composed of alkanes (77.6%), 12.4 wt% of benzene compounds, and about 9 wt% of naphthalene compounds. Diesel, also known as is made up of 23.6% aromatic compounds and 74% alkanes, as determined. With the use of Mn/Zn/AC hydroprocessing, our produced HPPO-B and diesel engines have an almost perfect match.

The alkanes that are included in the GC-MS information can be organised according to their carbon number range. PPO has alkanes that are roughly 9.2 weight percent in the range of C10–C15, 16 weight percent lies in the range of C16–C20, and 40 weight percent occurred in the range of C21–C25. The estimated weight percentage of alkanes in HPPO-B after hydrocracking is 23 weight percent in the C10–C15 range, 42 weight percent in the C16–C20 range, and 12 weight percent in the C21–C25 range. Regarding diesel, the alkane content ranges from approximately 26.5 weight percent in the C10–C15, 38.7 weight percent in the C16–C20, and 12.6 weight percent in the C21–C25. Considerable the hydrocracking process is produced by hydroprocessing because PPO contained 40% weight percentage of alkanes that in the range of C21–C25 before hydrocracking. It was discovered that HPPO-B's alkane makeup approximated that of the high-end commercialised diesel.

There are two types of alkanes found in commercial diesel: isoalkanes and alkanes with. In this the combustibility to burn properly, diesel must have an acceptable balance between isoalkanes to *n*-alkanes. Although *N*-alkanes have excellent cetane numbers, their cold flow performance is subpar. Aromatics have low cetane values but strong cold flow performance. The cetane and cold flow properties of isoalkanes are moderate, falling in the middle of those of aromatics and *n*-alkanes. Isoalkane formulation can function as a bridge to provide the required cetane and cold characteristics for flow.

The alkanes with found in PPO, commercial diesel and the synthesised HPPO-B are displayed in Fig. 5b. After hydroprocessing, all of the alkanes found in PPO were *n*-alkanes. In HPPO-B, the *n*-alkane content was roughly 17.2% in C10–C15, 26.2 wt% in C16–C20 line-up, and 12 wt% in C21–C25. About 16.5 weight percent of diesel is found in C10–C15 assortment, 24.2 weight percent in C16–C20, as well as 12.6 weight percent in C21–C25. The *n*-alkanes combination demonstrates an intact fit among diesel and HPPO-B.^58^

PPO had been found to have no isoalkanes, but HPPO-B created 21.8 weight percent isoalkanes by the hydroisomerization function. In contrast, commercial diesel had 24.8 weight percent isoalkanes. The isoalkanes exists in diesel and HPPO-B have been categorised according to the carbon number range in Fig. 5c. It was discovered that HPPO-B had isoalkanes of around 5.8 weight percent in C10–C15 and 16 weight percent in C16–C20. Isoalkane levels in superior commercial diesel were around 10.2 weight percent among those in C10–C15 besides 14.6 weight % in C16–C20.

PPO, superior diesel and the synthesised HPPO-B aromatic concentration is displayed in Fig. 5d. The aromatization enabled the production of about 23 weight percent of aromatic compounds in HPPO-B, whereas PPO only contained traces of aromatics. Heterocyclic content in diesel engines was determined to be 21.4 weight percent.

The corresponding carbon number range of the alkene compounds are shown in Fig. 5e and f. PPO's composition included about 33.6 weight percent alkenes are before hydroprocessing. The complete lack of alkenes in HPPO-B during hydroprocessing validated the efficiency of hydrogenation. According to GC-MS analyses of HPPO-B, PPO hydroprocessed with Mn/Zn/AC catalyst produced 95% of its identical composition to commercial diesel. It was discovered that hydroreforming LDPE possessed significant catalytic properties.^59,60^

## 4. Hydroprocessing's effectiveness

PPO has undergone hydroprocessing, which entails hydroisomerization, hydrogenation, and aromatization. The carbon chain ratios were used to analyse the hydroprocessing efficiency of the entire suite of functions. Considering the aforementioned ratio formulas, PPO and HPPO-B, the produced fuels, have been compared chemically to diesel.1
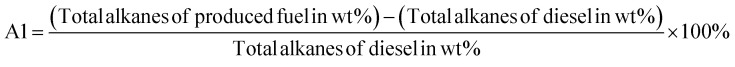


It became apparent that the total alkanes (A1) in PPO were 16.2% and 0.8% less in HPPO-B and diesel, respectively. Following treatment, there was a 99.2% match between the total alkanes in the commercial diesel and the synthesised HPPO-B. The most advantageous of diesel component compounds are alkanes, since they offer the cetane number required for fuel combustion and ignition timing. The accuracy with which the alkane content matches enhances HPPO-B's acceptability as a diesel engine fuel.2
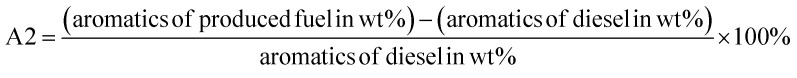
In PPO, which can total aromatics (A2) was found to be zero, however after treatment, HPPO-B had seven percent greater aromatic compounds than diesel.3
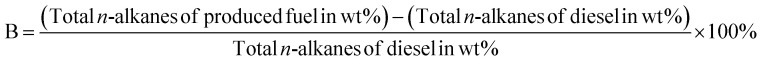


Subsequently was determined that PPO had a total of 22% higher *n*-alkanes (B) than diesel, in contrast, they were 3.7% more in HPPO-B compared to conventional petroleum products, implying that diesel and hydrocarbons matched 96.3 percent of the time that they existed.4
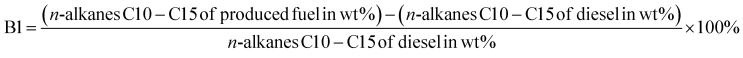


The fourth equation for *n*-alkanes in the C16–C20 and C21–C25 are indicated by the symbols B2 and B3, respectively.

Compared to diesel, PPO exhibited *n*-alkanes in carbon numbers such as C10–C15 which were 45% shorter in length whereas HPPO-B indicated 4.2% higher *n*-alkanes on behalf of the equivalent carbon number, culminating in a 95.6 percent concordance. While HPPO-B comprised an additional 9% of alkanes compared to diesel, resulting in a 91% coincide with diesel, PPO had 34% less *n*-alkanes within the C16–C20 carbon range of values. In the C21–C25 carbon range, PPO had 212% more *n*-alkanes than diesel, but HPPO-B coincided with diesel by 93.5%, having 6.5% fewer *n*-alkanes over diesel in the same carbon range. The vast majority of the PPO *n*-alkanes were found to be lies in carbon range of C21–C25 (212% compared to 19% in diesel), demonstrating that PPO is created with higher carbon alkanes than either HPPO-B or diesel-powered vehicles. Between C21 and C25 carbon, the alkane reduction from 212% to 6.5% confirms both the method and catalyst's the hydrocracking process efficacy.^61^5
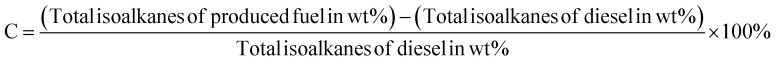


The design was determined that PPO's composition encompassed no total isoalkanes, and HPPO-B's composition contained 12% less isoalkanes compared to diesel, meaning that the two were 88% similar. HPPO-B's isoalkane content is raised through successful hydroisomerization made possible by the catalyst's characteristics and method.6
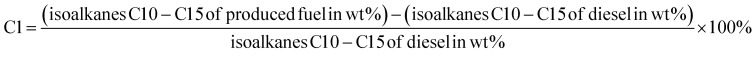


Exactly the power source C16–C20 range, C2 stands for the same eqn (6).

Further study has been done on the existence of isoalkanes at concentrations at carbon ranges of C10–C15 and C16–C20. Across the C10–C15, HPPO-B has forty-two percent fewer isoalkanes compared to diesel, while in the C16–C20, HPPO-B has 10% additional isoalkanes as opposed to diesel. The hydroisomerization intensification is required to achieve the diesel fuel objective. To equal diesel by in excess of 90 percent, the isoalkane concentration in C10–C15 needs to be increased.^62^

### 4.1. Engine combustion

#### 4.1.1. Peak pressure and rate of heat release in the cylinder

**Fig. 6:**
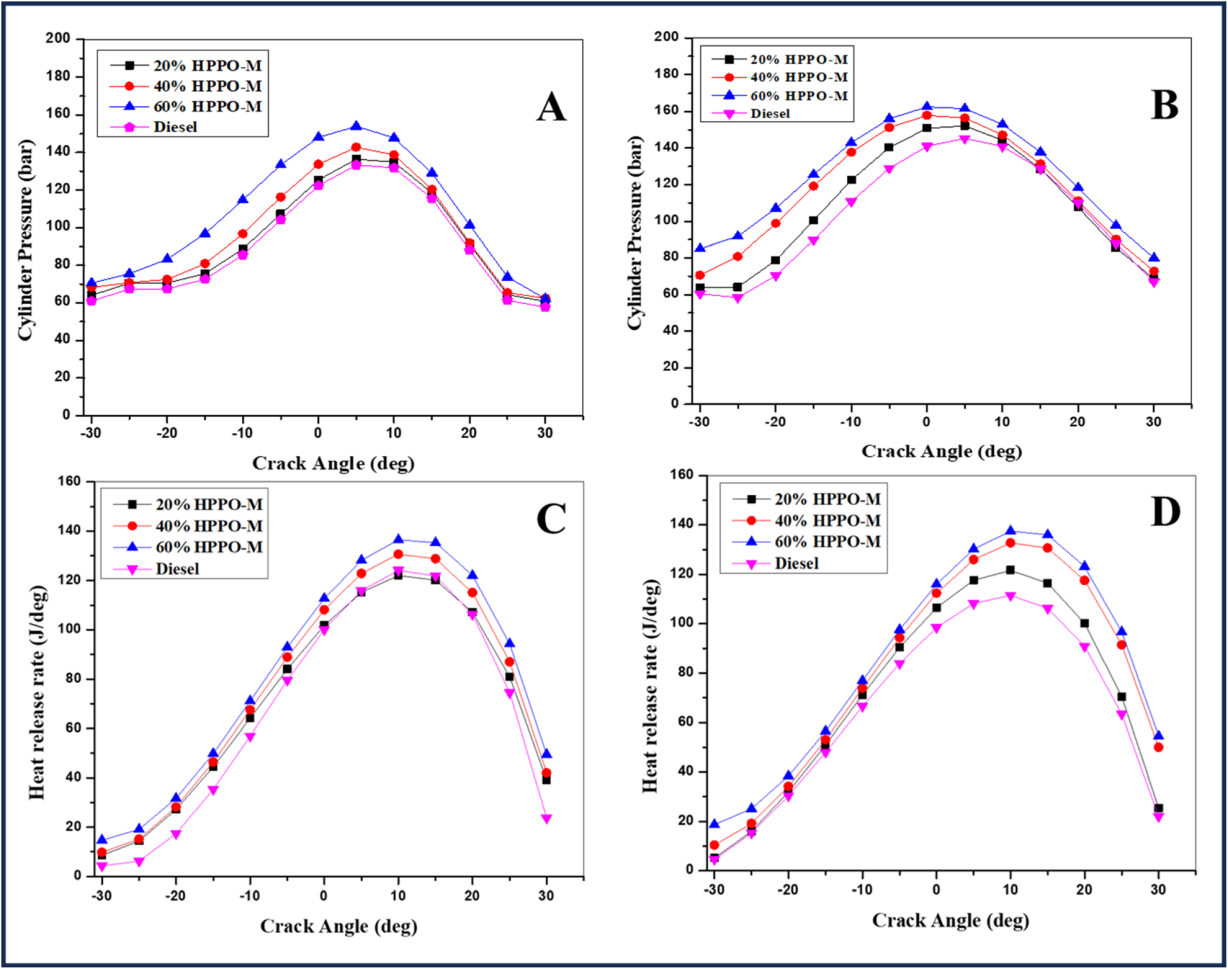
Cylinder pressure variation for (A) 100% load and (B) 75% load and variations in heating release rate (HRR) for (C) 100% load and (D) 75% load.

Fig. 6a and b, respectively, illustrate the values of the cylinder pressure during combustion at 100% and 75% load. When comparing HPPO-B to diesel, there were 2.5% to 3.7% less variances in 100% load peak pressure. Compared to diesel, the changes in peak pressure in the cylinders at 75% load were 2.6% to 3.9% smaller. The HPPO-B' maximum pressure performance was 96% of the values of commercial premium diesel. The hydrocracking process functionality is capable of raising the peak pressure performance to 100% compared to diesel values from the current 95%. Elevated pressure resulting from an extended lag interval period has been seen in prior PPO research. In HPPO-B, the alkenes conversion has reduced the ignition lag interval factor. Fig. 6c and d illustrate the HRR variance for the developed HPPO-B coordinates at 100% and 75% loading. At 100% load, it is discovered that the HRR variance in HPPO-B mixes ranges from 2.5% to 3.7% of the diesel value. It becomes apparent that the HRR variance for 75% load HPPO-B combinations obtained as 1.6% to 4.7% less than corresponding to premium diesel. It was shown that 95% concordance between the HRR outcomes for HPPO-B coordinates and diesel values was attained. When the process's hydrocracking efficiency rises from its present level of 95% to 100%, HPPO-B's HRR effectiveness will be reaching its best.^63^

#### 4.1.2. Engine performance

**Fig. 7:**
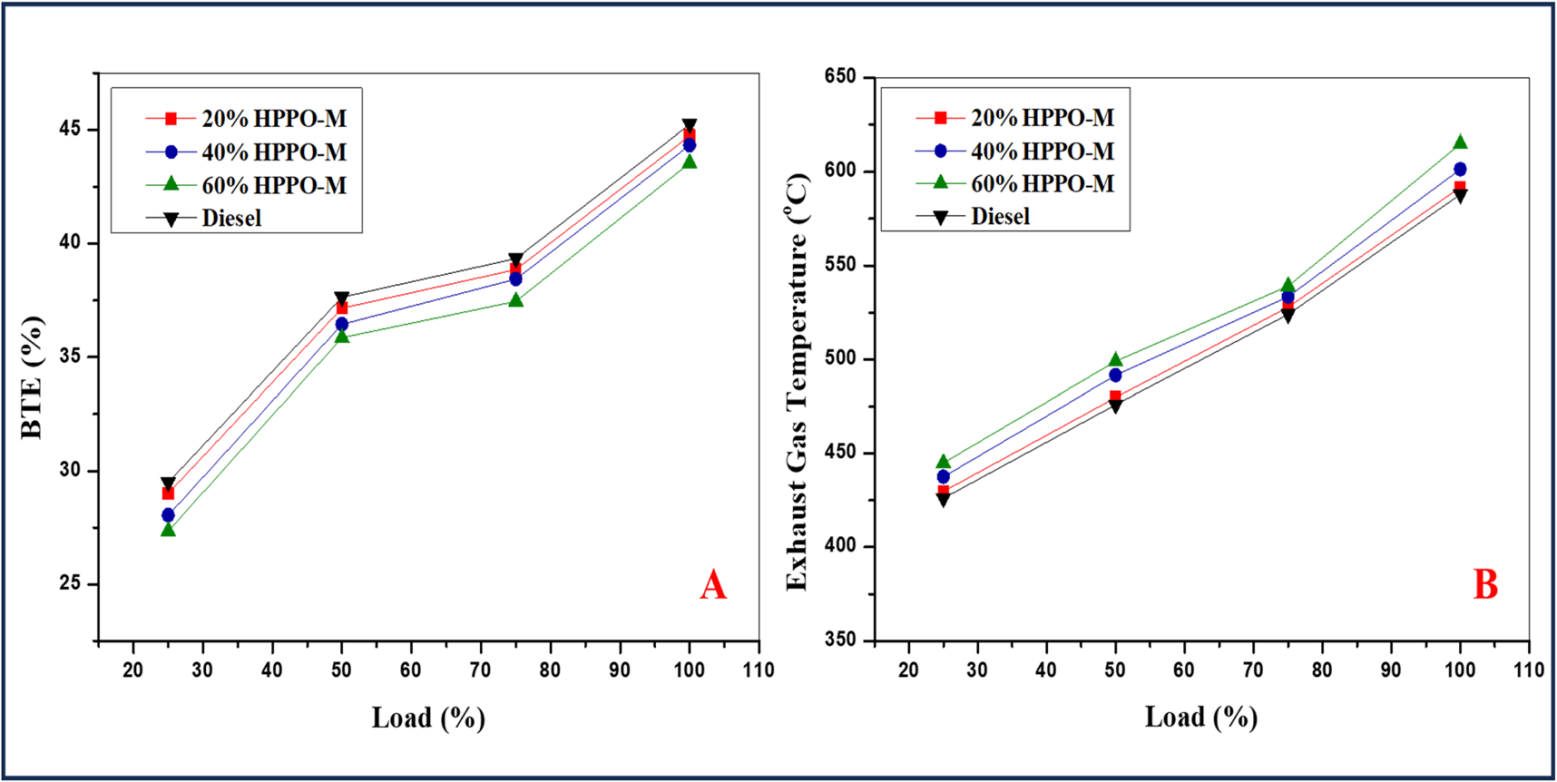
Variation in (A) BTE and (B) exhaust gas temperature.

When using fuel for generating work, the brake thermal efficiency (BTE) characteristics are implied by the percentage of net available output that results from a specific thermal input. The following describes the thermal measurement methodology which was used to determine work final product:7
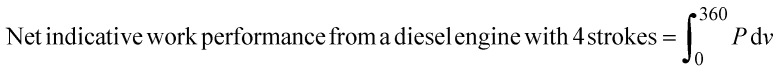
where *P* represents the chamber's pressure at the specified point during the cycle and d*v* is the variation in volume. The pressure variation in respect to the crank angle is the primary element influencing the process's output. The combustion pressure associated with HPPO-B has been determined to be 95% identical to that of diesel in Fig. 7a. As a result, it will be expected that the engine's total work performance at peak load will be equivalent to diesel values. Since commercial diesel and HPPO-B have the exact pumping losses, their impact on changes in output from the network is minimal. Although the manufactured HPPO-B's cetane index (CI) is higher compared to that of premium commercial diesel, the CI of the mixes intended is significantly higher than that of regular diesel. Better combustion will come from a combination of fuel with a greater amount of CI since it will ignite more quickly.

The variance in BTE (Brake thermal efficacy) for the HPPO-B mixture is displayed in The HPPO-B blend's fluctuation in BTE at 100% load was 1.2% and 4% less than diesel engines values. The previously HPPO-B blend's BTE variation at 75% load was 1.4% to a percentage of 6 lower than diesel values. The commercial regular diesel values were 93% of the BTE efficacy of the HPPO-B mix. According to earlier research by the pyrolysed crude oil BTE was lower compared to that of regular diesel. Less BTE for PPO is the result of increased thermal losses caused by a longer delay in ignition and the ensuing higher HRR.

The breakdown of BSFC (brake specific fuel consumption) variance for the regular diesel and HPPO-B is displayed in Fig. 7b. The HPPO-B blend's BSFC varied between 3% and 7.7% at 100% load, which was more than the diesel values. The range of BSFC for HPPO-B at 75% capacity was 1.4% to 7.5% higher compared to the equivalent amount of diesel. The temperature of the emitted gas variation of the regular diesel and HPPO-B is shown in Fig. 7c. Compared to premium commercial diesel, the temperature variance of emitted gas for HPPO-B was 2.8% to 8% greater at 100% load. At a load of 75%, the temperature variance of engine emitted gas associated with the HPPO-B was 2.5% to 7.5% more than the variation seen with the diesel.

#### 4.1.3. Engine emission analysis

**Fig. 8:**
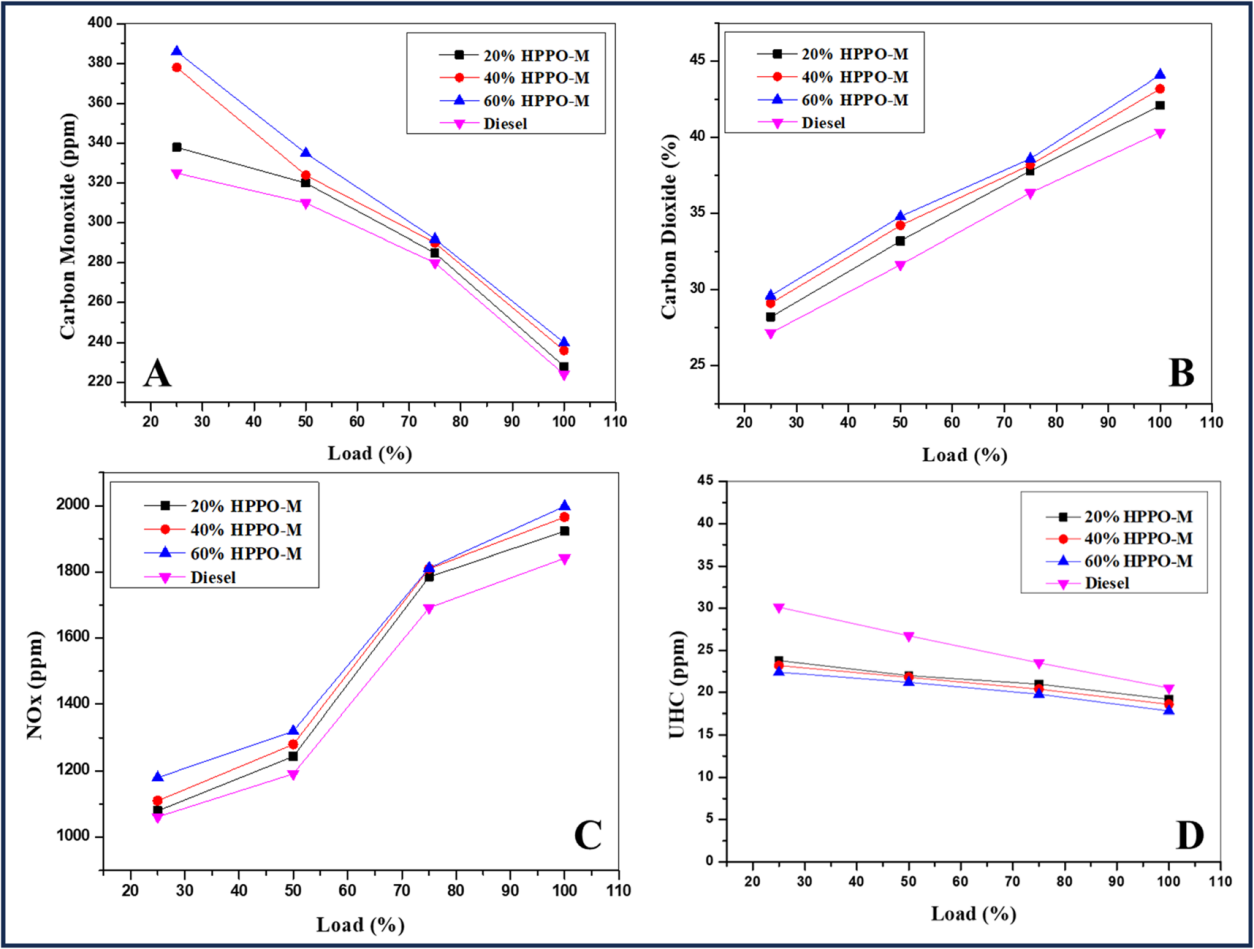
Variation of (A) CO with load, (B) CO_2_ with load, (C) NOx with load and (D) HC with load.

The variance in HPPO-B carbon monoxide (CO) gas emissions that have been established is shown in Fig. 8a. Diesel has been determined to have CO discharge levels of 30 ppm at a load of 100%, while HPPO-B showed a variance of four parts per million (ppm) to 9 ppm higher compared to regular diesel, comparable to the diesel values. Diesel was found to have greenhouse gas emissions of 82 ppm at a 75% load, while the CO emissions of HPPO-B mixes varied between 8 and 18 ppm above that of diesel. At greater loads, HPPO-B's CO emissions were more similar to those of diesel; nevertheless, at minimal temperatures, it was observed as 11–36 ppm improved in order to regular diesel. The end outcome of not having enough oxidizer for burning is CO petrol emissions. When the proportion of carbon-based fuels increases, CO emissions are likewise predicted to rise. As previously mentioned, larger blending ratios lead to higher emissions of CO. Prior research on additive-based pyrolysis oil has demonstrated reduced emissions of carbon dioxide.

The HPPO-B blend's change in carbon dioxide (CO_2_) emission levels is shown in Fig. 8b. The variance in HPPO-B, CO_2_ exhaustion was noted as 2.4% to 8.7% greater at the full load level and at 75% load it was maintained as 3.1% to 8.4% compared to the regular diesel. The amount of carbon that is present in HPPO-B's composition determines the amount of CO_2_ emissions. The alkane composition matches diesel values by around 95%. If hydroprocessing the same material with 100% efficiency is probable, subsequently CO and CO_2_ discharges ought to correlate with regular diesel by 100%.

The HPPO-B and commercial regular diesel engine's NOx exhaustion limits vary with the applied load, as shown in Fig. 8c. The NOx variation of the HPPO-B mix at 100% load varied from 0.6% to 4.5% relative to superior commercial-use diesel values. The HPPO-B's NOx at 75% load was 1.8% to 4.4% greater compared to the equivalent of superior commercial use diesel. HPPO-B blend's NOx emission levels matched diesel perfectly. According, diesel emits 40% less NOx than petrol. An increase in NOx for pyrolysis oil was previously noted. The kinetics of fuel reactions have a connection to emissions through a prompt process for NOx generation, in which carbon compounds function as facilitators. The mechanism of NOx generation is explained by the reaction listed below.

8 CH + N_2_ ↔ HCN + N

9 C + N ↔ CN + N

The design can be inferred from formulations that result in increased NOx emissions from fuels with higher carbon content. The Zeldovich mechanism states that increasing exhaust temperature and HRR promote the thermal exhaustion of NOx. The NOx emission levels of HPPO-B are likewise within diesel limits, given that its carbon number values and HRR are more comparable to those of diesel.

The increase or decrease of UHC (unburned hydrocarbon) exhaustion of HPPO-B and premium commercial use diesel with applied load is shown in Fig. 8d. The range in HPPO-B UHC at 100% load was 1.8% to 9.1% fewer comparable to the regular diesel values. The HPPO-B blend's UHC varied between 1.6% and 9.5% minimal in accordance with 75% load diesel values. The HPPO-B manufacturing as a substitute diesel engine fuel was successful since its UHC emission levels were fewer in order to diesel. Prior research explored that with higher combinations, oil used in plastic pyrolysis produced more UHC emissions.

#### 4.1.4. Catalytic activity of hydroprocessing Mn/Zn-AC

Furthermore, generated HPPO-B's composition, combustion, emissions, physical and chemical characteristics have been compared with those of HPPO which is created by hydrogenating PPO with mono metallic Ni/ZSM5. The physical–chemical specifications of HPPO and HPPO-B, both met EN590 requirements and produced comparable results. When compared to standard HPPO, the chemical constitution of HPPO-B showed an important enhancement. On comparison of the hydrogenation and the hydrocracking process functionalities attained in HPPO, the Mn/Zn-AC catalyst produced hydroisomerization, hydrogenation, aromatization, and hydrocracking into activities. In HPPO-B, aromatization resulted in an aromatic concentration that was 7% greater than diesel; in HPPO, perhaps there were merely tiny portions of aromatic components were identified. The Mn/Zn-AC catalyst yielded isoalkanes and *n*-alkanes in a ratio of 2.8 and for *n*-alkanes to isoalkanes in the ratio of 2.2 was found in diesel. In HPPO-B, the bimetallic catalyst was capable to create *n*-alkanes, isoalkanes, and aromatics in amounts that were more akin to the chemical structure of diesel. HPPO-B produced better combustion and emission results due to the fact the way its physical properties and chemical structure match for diesel.

The HPPO-B combustion peak pressure exhibited a maximum fluctuation of 4%, demonstrating a lower value than diesel. In the prior investigation, it was noted that for a 40% blend, HPPO generated a peak pressure that was approximately 15% greater than diesel. The greatest departure from the average HRR for the HPPO-B was 4.7% less comparable with the regular diesel value, whereas the maximum deviation for HPPO remained 15% greater in comparison with the diesel engines. The HPPO-B's BTE generated the largest variations, which was 6.45% of the total less than that of diesel. HPPO's BTE measurements for the 40% blend were sixteen percent lower than diesel's. The extreme BSFC variation of HPPO-B was seven percent greater with that of commercial diesel. The largest fluctuation in the amount that was used of BSFC for HPPO was found to amount to sixteen percentile points more than the amount found in diesel for a 30% blend.

The two hydroprocessed polypropylene pyrolysis oil and hydroprocessed polypropylene pyrolysis oil had comparable CO emissions at maximum loads; however, at lower loads, HPPO-B's emissions were more akin to diesel engines than HPPO's. At lower loads, HPPO-B achieved a maximum CO divergence of 36 ppm above commercially diesel, and HPPO had a minimum CO deviation of 75 ppm above commercial diesel. The CO_2_ emissions that resulted from the hydroprocessed polypropylene pyrolysis oil combine it with were found to be 8.7% higher than those from diesel, and the CO_2_ concentration of the HPPO blend was 40% greater in comparison to that of diesel. While the forty per cent blend of HPPO indicated a forty percentage point rise in NOx over diesel, the NOx for hydroprocessed polypropylene pyrolysis oil was 5.2% higher than diesel. While HPPO-B exhibited UHC emissions 11% higher than diesel, HPPO had UHC emissions 12% fewer than commercial use diesel. The performance of the HPPO at 10 and 20 weight percent were more similar to diesel; however, the at both thirty and forty weight percent displayed a notable divergence. The fuel efficiency, combustion, composition, and exhaustion of hydroprocessed polypropylene pyrolysis oil were substantially enhanced by the Mn/Zn-AC catalyst in all.

## 5. Conclusion

Hydroprocessed Polypropylene Pyrolysis Oil (HPPO-B) is a renewable diesel alternative produced using a Mn/Zn-AC bimetallic catalyst to hydroprocess polypropylene pyrolysis oil (PPO). The resulting HPPO-B closely matches the physical, chemical, and performance characteristics of premium commercial diesel, complying with European EN590 standards, including viscosity (3.9 mm^2^ s^−1^), density (855 kg m^−3^), and a cetane index of 64. GCMS analysis revealed complete hydrogenation, transforming alkenes into alkanes and achieving a 95% match with diesel's *n*-alkane composition across C10–C15, C16–C20, and C21–C25 carbon chains. HPPO-B contained 7% more aromatics than diesel due to aromatization, while hydroisomerization resulted in 21.8% isoalkanes compared to diesel's 24.8%, with isoalkane levels in the C10–C15 range 43% lower than diesel, requiring further catalyst optimization. Combustion performance showed a 95% match in heat release rates (HRR) and a 96% match in peak pressure with diesel, with emissions of CO, CO_2_, and NOx remaining comparable. Additionally, HPPO-B demonstrated 10% lower unburned hydrocarbon (UHC) emissions and achieved 93% and 92% matches in brake-specific fuel consumption (BSFC) and brake thermal efficiency (BTE), respectively. HPPO-B offers a sustainable solution for recycling post-consumer polypropylene plastic waste into high-quality fuel, with a maximum blending ratio of 40% in commercial diesel engines. Future research should focus on enhancing the Mn/Zn-AC catalyst to improve isoalkane content, reduce aromatics, increase blending ratios, and scale the process for industrial applications while conducting long-term engine durability tests and lifecycle assessments to evaluate environmental impacts.

## Data availability

Characterization data along with further supporting data referenced in the manuscript are available in the ESI.†

## Conflicts of interest

There are no conflicts to declare.

## Supplementary Material

RA-015-D5RA00082C-s001

## References

[cit1] Sharuddin S. D. A., Abnisa F., Daud W. M. A. W., Aroua M. K. (2016). A review on pyrolysis of plastic wastes. Energy Convers. Manag..

[cit2] Murugan S., Ramaswamy M. C., Nagarajan G. (2008). The use of tyre pyrolysis oil in diesel engines. Waste Manag..

[cit3] Palos R., Gutierrez A., Vela F. J., Olazar M., Arandes J. M., Bilbao J. (2021). Waste refinery: the valorization of waste plastics and end-of-life tires in refinery units. A review. Energy Fuels.

[cit4] Speight J. G. , The Refinery of the Future, Gulf Professional Publishing, 2020

[cit5] Qureshi M. S., Oasmaa A., Pihkola H., Deviatkin I., Tenhunen A., Mannila J. (2020). *et al.*, Pyrolysis of plastic waste: Opportunities and challenges. J. Anal. Appl. Pyrolysis.

[cit6] Bezergianni S., Dimitriadis A., Faussone G.-C., Karonis D. (2017). Alternative diesel from waste plastics. Energies.

[cit7] Farooqui S. A. , Kumar R., Sinha A. K. and Ray A., Green diesel production by hydroprocessing technology, in Green Diesel: an Alternative to Biodiesel and Petrodiesel, Advances in Sustainability Science and Technology, ed. M. Aslam, S. Shivaji Maktedar and A. K. Sarma, Springer, Singapore, 2022, 10.1007/978-981-19-2235-0_4

[cit8] Malpani S. K. , Hada R., Kumar A. and Goyal D., Waste-derived activated carbon as a sustainable and economical catalyst support, in Handbook of Porous Carbon Materials. Materials Horizons: from Nature to Nanomaterials, ed. A. N. Grace, P. Sonar, P. Bhardwaj and A. Chakravorty, Springer, Singapore, 2023, 10.1007/978-981-19-7188-4_13

[cit9] Wang Q., Lu J., Liu S., Yu B., Liang B. (2024). Nanopolyhedral Zn/Fe-NC derived from bimetallic zeolitic imidazole frameworks as an efficient catalyst for the oxygen reduction reaction in an air-cathode microbial fuel cell. New J. Chem..

[cit10] Majodina S., Poswayo O., Dembaremba T. O., Tshentu Z. R. (2023). Towards improvement of hydroprocessing catalysts - understanding the role of advanced mineral materials in hydroprocessing catalysts. Miner. Mater..

[cit11] Krutof A., Hawboldt K. (2016). Blends of pyrolysis oil, petroleum, and other bio-based fuels: A review. Renew. Sustain. Energy Rev..

[cit12] Atmanli A., Yilmaz N. (2018). A comparative analysis of n-butanol/diesel and 1-pentanol/diesel blends in a compression ignition engine. Fuel.

[cit13] Atmanli A., Yilmaz N. (2020). An experimental assessment on semi-low temperature combustion using waste oil biodiesel/C3-C5 alcohol blends in a diesel engine. Fuel.

[cit14] Yilmaz N., Davis S. M. (2016). Polycyclic aromatic hydrocarbon (PAH) formation in a diesel engine fueled with diesel, biodiesel and biodiesel/n-butanol blends. Fuel.

[cit15] Yilmaz N., Atmanli A., Vigil F. M. (2018). Quaternary blends of diesel, biodiesel, higher alcohols and vegetable oil in a compression ignition engine. Fuel.

[cit16] Atmanli A. (2020). Experimental comparison of biodiesel production performance of two different microalgae. Fuel.

[cit17] Akpanudoh N. S., Gobin K., Manos G. (2005). Catalytic degradation of plastic waste to liquid fuel over commercial cracking catalysts: effect of polymer to catalyst ratio/acidity content. J. Mol. Catal. A Chem..

[cit18] Mangesh V. L., Padmanabhan S., Tamizhdurai P., Ramesh A. (2020). Experimental investigation to identify the type of waste plastic pyrolysis oil suitable for conversion to diesel engine fuel. J. Clean. Prod..

[cit19] Mangesh V. L., Tamizhdurai P., Umasankar S., Palaniswamy R., Narayanan S., Augustine T. (2022). *et al.*, Hydroprocessing mixed waste plastics
to obtain clean transport fuel. J. Clean. Prod..

[cit20] Hashim H., Narayanasamy M., Yunus N. A., Shiun L. J., Ab Muis Z., Ho W. S. (2017). A cleaner and greener fuel: Biofuel blend formulation and emission assessment. J. Clean. Prod..

[cit21] Bezergianni S., Dimitriadis A. (2013). Comparison between different types of renewable diesel. Renew. Sustain. Energy Rev..

[cit22] Rowley J. R., Freeman D. K., Rowley R. L., Oscarson J. L., Giles N. F., Wilding W. V. (2010). Flash point: evaluation, experimentation and estimation. Int. J. Thermophys..

[cit23] Aboul-Gheit A. K., Gad F. K., Abdel-Aleem G. M., El-Desouki D. S., Abdel-Hamid S. M., Ghoneim S. A., Ibrahim A. H. (2014). Pt, Re and Pt–Re incorporation in sulfated zirconia as catalysts for n-pentane isomerization. Egypt. J. Pet..

[cit24] Busto M., Vera C. R., Grau J. M. (2011). Optimal process conditions for the isomerization–cracking of long-chain n-paraffins to high octane isomerizate gasoline over Pt/SO42–ZrO2 catalysts. Fuel Process. Technol..

[cit25] Stichert W., Schüth F. (1998). Synthesis of catalytically active high surface area monoclinic sulfated zirconia. J. Catal..

[cit26] Gu L., Chen X., Zhou Y., Zhu Q., Huang H., Lu H. (2017). Propene and CO oxidation on Pt/Ce-Zr-SO42–diesel oxidation catalysts: Effect of sulfate on activity and stability. Chin. J. Catal..

[cit27] Rachmat A., Trisunaryanti W., Sutarno W. K. (2017). Synthesis and characterization of sulfated zirconia mesopore and its application on lauric acid esterification. Mater. Renew. Sustain. Energy.

[cit28] Sing K. S. (1985). Reporting physisorption data for gas/solid systems with special reference to the determination of surface area and porosity (Recommendations 1984). Pure Appl. Chem..

[cit29] Thommes M., Kaneko K., Neimark A. V., Olivier J. P., Rodriguez-Reinoso F., Rouquerol J., Sing K. S. (2015). Physisorption of gases, with special reference to the evaluation of surface area and pore size distribution (IUPAC Technical Report). Pure Appl. Chem..

[cit30] Sekewael S. J., Pratika R. A., Hauli L., Amin A. K., Utami M., Wijaya K. (2022). Recent progress on sulfated nanozirconia as a solid acid catalyst in the hydrocracking reaction. Catalysts.

[cit31] Hauli L. A. T., Wijaya K., Syoufian A. K. (2019). Hydrocracking of LDPE plastic waste into liquid fuel over sulfated zirconia from a commercial zirconia nanopowder. Orient. J. Chem..

[cit32] Heshmatpour F., Aghakhanpour R. B. (2012). Synthesis and characterization of superfine pure tetragonal nanocrystalline sulfated zirconia powder by a non-alkoxide sol–gel route. Adv. Powder Technol..

[cit33] Fu B., Gao L., Niu L., Wei R., Xiao G. (2009). Biodiesel from waste cooking oil via heterogeneous superacid catalyst SO42−/ZrO2. Energy Fuels.

[cit34] Kaur N., Ali A. (2015). Preparation and application of Ce/ZrO2− TiO2/SO42− as solid catalyst for the esterification of fatty acids. Renew. Energy.

[cit35] La Ore M. S., Wijaya K., Trisunaryanti W., Saputri W. D., Heraldy E., Yuwana N. W. (2020). *et al.*, The synthesis of SO4/ZrO2 and Zr/CaO catalysts via hydrothermal treatment and their application for conversion of low-grade coconut oil into biodiesel. J. Environ. Chem. Eng..

[cit36] Fayad G. (2023). *et al.*, Hydroprocessing of plastic pyrolysis oils using Ni/ZSM-5 catalysts: Effects on fuel properties and emissions. Energies.

[cit37] Utami M., Trisunaryanti W., Shida K., Tsushida M., Kawakita H., Ohto K. (2019). *et al.*, Hydrothermal preparation of a platinum-loaded sulphated nanozirconia catalyst for the effective conversion of waste low-density polyethylene into gasoline-range hydrocarbons. RSC Adv..

[cit38] Zhang L., Song Y., Guo W., Zhao J. (2022). Preparation of activated carbon from facial mask waste and its application for pollutant removal. J. Environ. Chem. Eng..

[cit39] Kumar P., Kumar A. (2021). Pyrolysis of waste materials for the production of activated carbon: A review. Renew. Sustain. Energy Rev..

[cit40] Feng Y., Wu Y., Wang W. (2020). Characterization of activated carbon from waste textiles and its application in water treatment. J. Environ. Manage..

[cit41] Marcilla A., Gomez A., Reyes-Labarta J. A., Giner A. (2003). Catalytic pyrolysis of polypropylene using MCM-41: Kinetic model. Polym. Degrad. Stab..

[cit42] Qureshi M. S., Oasmaa A., Pihkola H., Deviatkin I., Tenhunen A., Mannila J. (2020). *et al.*, Pyrolysis of plastic waste: Opportunities and challenges. J. Anal. Appl. Pyrolysis.

[cit43] Hauli L., Wijaya K., Armunanto R. (2018). Preparation and characterization of sulfated zirconia from a commercial zirconia nanopowder. Orient. J. Chem..

[cit44] Wu C. H., Chang C. Y., Hor J. L., Shih S. M., Chen L. W., Chang F. W. (1993). On the thermal treatment of plastic mixtures of MSW: Pyrolysis kinetics. Waste Manag..

[cit45] Ranzi E., Dente M., Faravelli T., Bozzano G., Fabini S., Nava R. (1997). *et al.*, Kinetic modeling of polyethylene and polypropylene thermal degradation. J. Anal. Appl. Pyrolysis.

[cit46] Friedman H. L. (1964). Kinetics of thermal degradation of char-forming plastics from thermogravimetry. Application to a phenolic plastic. J. Polym. Sci., Part C.

[cit47] Mftah A., Alhassan F. H., Al-Qubaisi M. S., El Zowalaty M. E., Webster T. J., Sh-Eldin M. (2015). *et al.*, Physicochemical properties, cytotoxicity, and antimicrobial activity of sulphated zirconia nanoparticles. Int. J. Nanomed..

[cit48] Utami M., Wijaya K., Trisunaryanti W. (2018). Pt-promoted sulfated zirconia as catalyst for hydrocracking of LDPE plastic waste into liquid fuels. Mater. Chem. Phys..

[cit49] Said A. E. A., Abd El-Wahab M. M., Abd El-Aal M. (2014). The catalytic performance of sulfated zirconia in the dehydration of methanol to dimethyl ether. J. Mol. Catal. A Chem..

[cit50] Al-Salem S. M., Lettieri P. (2010). Kinetic study of high-density polyethylene (HDPE) pyrolysis. Chem. Eng. Res. Des..

[cit51] Dubdub I., Al-Yaari M. (2021). Thermal behavior of mixed plastics at different heating rates: I. pyrolysis kinetics. Polymers.

[cit52] Flynn J. H., Wall L. A. (1966). A quick, direct method for the determination of activation energy from thermogravimetric data. J. Polym. Sci., Part B..

[cit53] Briceno J., Lemos M. A., Lemos F. (2021). Kinetic analysis of the degradation of HDPE+PP polymer mixtures. Int. J. Chem. Kinet..

[cit54] Hujuri U., Ghoshal A. K., Gumma S. (2008). Modeling pyrolysis kinetics of plastic mixtures. Polym. Degrad. Stab..

[cit55] Jameel A. G. A., Han Y., Brignoli O., Telalović S., Elbaz A. M., Im H. G. (2017). *et al.*, Heavy fuel oil pyrolysis and combustion: Kinetics and evolved gases investigated by TGA-FTIR. J. Anal. Appl.
Pyrolysis.

[cit56] Rizzo A. M., Chiaramonti D. (2022). Blending of Hydrothermal Liquefaction Biocrude with Residual Marine Fuel: An Experimental Assessment. Energies.

[cit57] Adams J., Bornstein J. M., Munno K., Hollebone B., King T., Brown R. S. (2014). *et al.*, Identification of compounds in heavy fuel oil that are chronically toxic to rainbow trout embryos by effects-driven chemical fractionation. Environ. Toxicol. Chem..

[cit58] Lonyi F., Valyon J. (1996). Thermally effected structural and surface transformations of sulfated TiO2, ZrO2 and TiO2-ZrO2 catalysts. J. Therm. Anal. Calorim..

[cit59] Song Y., Tian J., Ye Y., Jin Y., Zhou X., Wang J. A. (2013). *et al.*, Effects of calcination temperature and water-washing treatment on n-hexane hydroisomerization behavior of Pt-promoted sulfated zirconia based catalysts. Catal. Today.

[cit60] Ren K., Kong D., Meng X., Wang X., Shi L., Liu N. (2019). The effects of ammonium sulfate and sulfamic acid on the surface acidity of sulfated zirconia. J. Saudi Chem. Soc..

[cit61] Mangesh V. L., Tamizhdurai P., Krishnan P. S., Narayanan S., Umasankar S., Padmanabhan S. (2020). *et al.*, Green energy: Hydroprocessing waste polypropylene to produce transport fuel. J. Clean. Prod..

[cit62] Thian Tye C. , Catalysts for Hydroprocessing of Heavy Oils and Petroleum Residues, IntechOpen, 2019, 10.5772/intechopen.89451

[cit63] Mangesh V. L., Perumal T., Subramanian S., Padmanabhan S. (2020). Clean energy from plastic: Production of hydroprocessed waste polypropylene pyrolysis oil utilizing a Ni–Mo/laponite catalyst. Energy Fuels.

