# Supplementary material for: Green energy production: hydroprocessing of waste plastic to diesel fuel using bimetal of Mn/Zn supported on activated carbon
Source: RSC Adv. 2025 Mar 11;15(10):7769–85. doi: 10.1039/d5ra00082c (PMC11895529; doi:10.1039/d5ra00082c)
Supplement: RA-015-D5RA00082C-s001 [file RA-015-D5RA00082C-s001.pdf]

This journal is © The Royal Society of Chemistry 2025

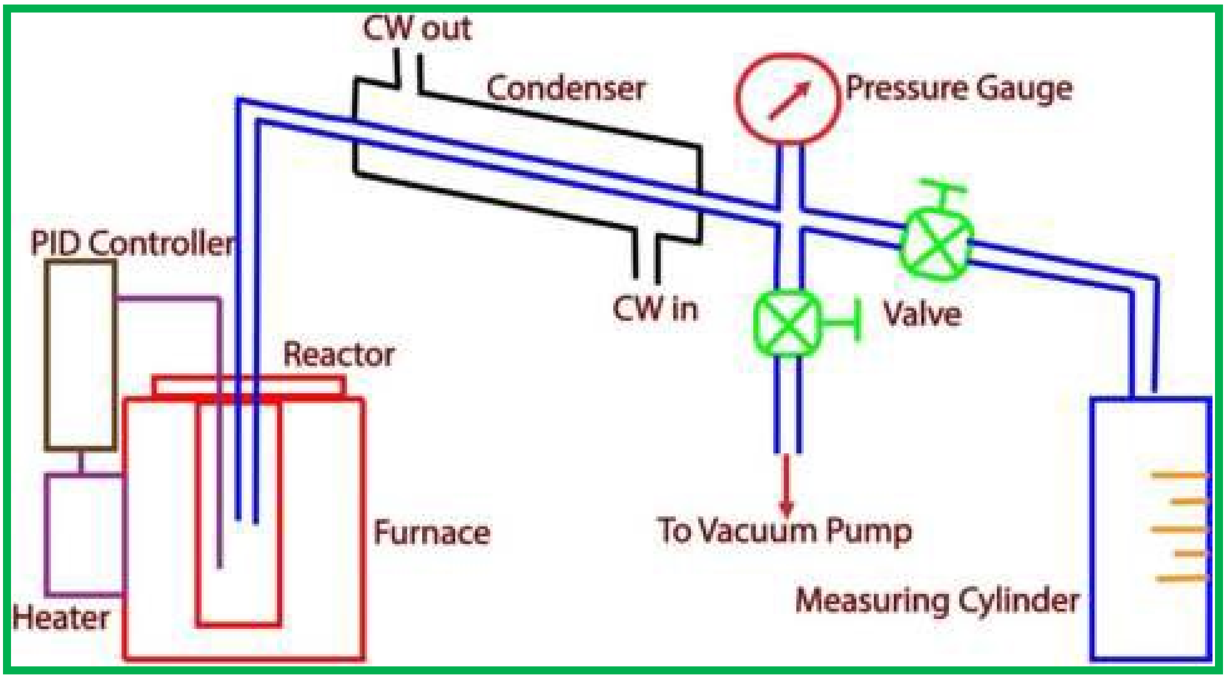

Figure S1 - Pyrolysis Reactor

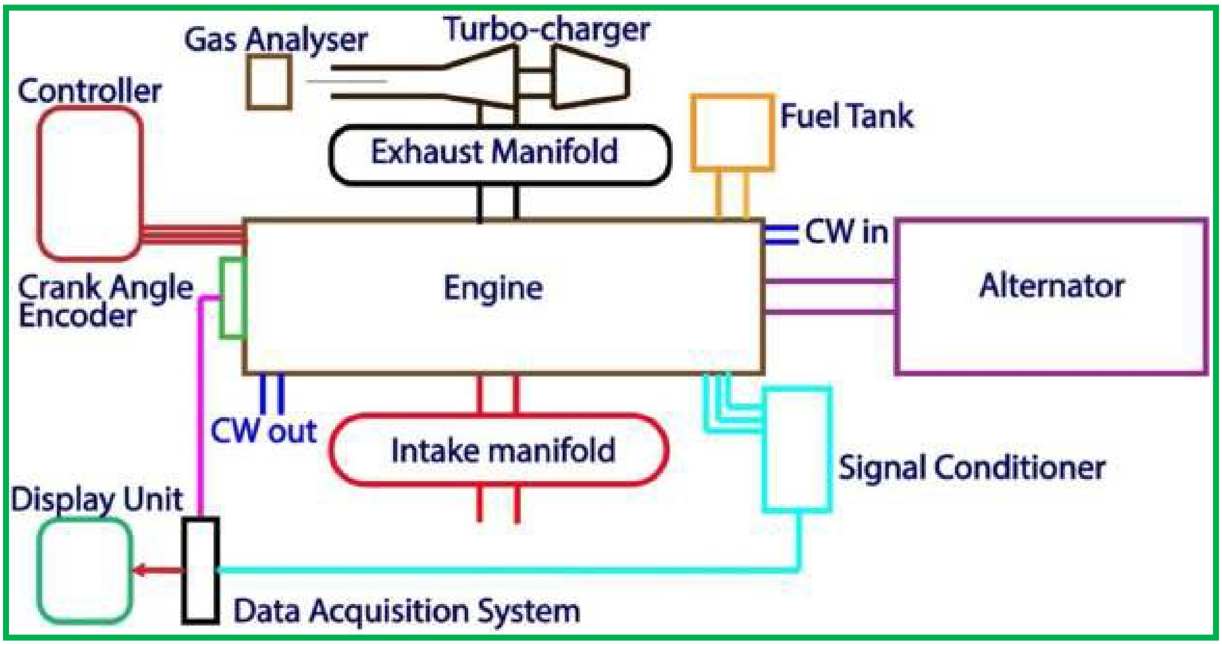

Figure S2 - Diesel engine carried out

Table 1. Engine Specifications

**Engine Brand**	**Eicher E483**
**Diesel Engine**	Type 4 cylinder in-line T/C, DI
**Compression ratio**	18.6:2
**Displacement**	3600 cm^3
**Max. power**	70 kW
**Turbocharger**	Boost pressure
**Injector: hole _ dia**	5 _ 0.209 mm
**Injection timing**	15 °CA btdc
**Nozzle opening pr**	25 MPa
**Bore × Stroke**	105 mm × 110mm

Table.2. Gas Chromatography Mass Spectrometry (GC-MS) of Mixed Plastic Pyrolysis Oil (MPPO)

S.No	**Retention Time**	**Compound Name**	**Chemical Formula**	**% Area**
**1**	5.6	Benzene, (1-methyethy)-	C9H12	0.57
**2**	6.1	1-Decene	C10H20	2.19
**3**	6.7	Decane	C10H22	1.11
**4**	7.2	1-Undecene	C11H22	1.17
**5**	7.3	Undecane	C11H24	0.86
**6**	7.8	1-Dodecene	C12H24	1.23
**7**	7.9	Dodecane	C12H26	2.91
**8**	7.8	1-Tridecene	C13H26	2.31
**9**	7.9	Tridecane	C13H28	1.01
**10**	8.6	3-Tetradecene,(E)-	C14H28	1.57
**11**	8.7	2-Tetradecene,(E)-	C14H28	1.16
**12**	9.6	Tetradecane	C14H30	2.35
**13**	9.7	5-Tetradecene,(E)-	C14H28	1.07
**14**	9.8	1-Pentadecene	C15H30	1.82
**15**	10.1	Pentadecane	C15H32	0.86
**16**	11.3	5-Octadecene,(E)-	C18H36	1.38

**17**	11.5	7-Hexadecene,(Z)-	C16H32	1.07
**18**	12.1	Hexadecane	C16H34	1.86
**19**	12.4	8-Heptadecene	C17H34	1.46
**20**	12.5	1-Heptadecene	C17H34	2.29
**21**	12.6	Heptadecane	C17H36	0.7
**22**	13.7	1-Octadecene	C18H36	1.27
**23**	14.6	Octadecane	C18H38	1.45
**24**	14.8	1-Nonadecene	C19H38	2.51
**25**	16.4	Heptadecane	C17H36	0.81
**26**	17.3	1-Nonadecene	C19H38	1.66
**27**	17.5	Eicosane	C20H42	2.47
**28**	17.6	1-Nonadecene	C19H38	1.19
**29**	19.5	Heneicosane	C21H44	0.46
**30**	19.9	9-Nonadecene	C19H38	3.84
**31**	21.6	Eicosane	C20H42	0.83
**32**	21.7	9-Tricosene,(Z)-	C23H46	0.92
**33**	22.1	Eicosane	C20H42	2.57
**34**	24.2	1-Docosene	C22H44	0.79
**35**	24.3	Tetracosane	C24H50	2.78
**36**	26.3	9-Tricosene,(Z)-	C23H46	3.88
**37**	28.1	Pentacosane	C25H52	3.4
**38**	30.2	9-Tricosene,(Z)-	C23H46	3.83
**39**	31.7	Tetracosane	C24H50	3.32
**40**	33.6	9-Tricosene,(Z)-	C23H46	3.85
**41**	35.2	Tetracosane	C24H50	4.74
**42**	36.5	Tetracosane	C24H50	3.26
**43**	38.3	Tetracosane	C24H50	4.32
**44**	40.4	Eicosane	C20H42	2.89
**45**	42.1	Heneicosane	C21H44	1.62
**46**	44.3	Tetracosane	C24H50	3.13
**47**	46.3	Heneicosane	C21H44	2.53
**48**	48.3	Eicosane	C20H42	2.83
**49**	50.2	Tetracosane	C24H50	1.66

Table S3. Gas Chromatography Mass Spectrometry (GC-MS) of HPPO-M

**S.No**	**Retention Time**	**Compound Name**	**Chemical Formula**	**% Area**
**1**	5.4	Benzene, propyl	C9H12	2.37
**2**	5.6	Benzene, 1-ethyl-3-methyl-	C9H12	5.25
**3**	5.8	Benzene, 1,2,3-trimethyl-	C9H12	4.77

**4**	6.1	Benzene, 1,2,4-trimethyl-	C9H12	8.28
**5**	6.5	Benzene, 1,2,4-trimethyl-	C9H12	5.56
**6**	7.1	Benzene, 1-methyl-3-propyl	C10H14	2.96
**7**	7.2	Benzene, 1-ethyl-3,5-dimethyl-	C10H14	3.40
**8**	7.3	Benzene, 1-methyl-2-propyl-	C10H14	1.39
**9**	7.4	Benzene, 1-ethyl-2,4-dimethyl	C10H14	2.40
**10**	7.5	Benzene, 4-ethyl-1,2-dimethyl-	C10H14	2.78
**11**	7.8	Undecane	C11H24	1.79
**12**	8.2	Benzene, 1,2,4,5-tetramethyl-	C10H14	2.73
**13**	8.1	Undecane,3-methyl-	C12H26	3.80
**14**	8.8	1H-indene	C10H14	2.76
**15**	9.4	Undecane,3-methyl-	C12H26	2.35
**16**	9.8	Napthalene	C10H8	2.42
**17**	10.1	Dodecane	C12H26	3.15
**18**	11.3	1H-indene	C13H28	1.54
**19**	11.7	Napthalene,1,2,3,4-tetrahydro-6-dimethyl	C11H14	1.33
**20**	12.4	Napthalene, 2-methyl-	C11H10	1.37
**21**	12.6	Tridecane	C13H28	2.69
**22**	14.5	Dodecane, 2,6,10-trimethyl-	C15H32	1.46
**23**	14.8	Napthalene,1,2,3,4-tetrahydro-6-dimethyl	C11H14	1.31
**24**	15.2	Tetradecane	C14H30	3.38
**25**	16.7	Hexadecane	C16H34	1.94
**26**	17.7	Pentadecane	C15H32	3.20
**27**	18.6	Tridecane, 2,5-dimethyl	C15H32	1.14
**28**	19.1	Pentadecane, 2-methyl-	C16H34	0.18
**29**	19.8	Hexadecane	C16H34	2.80
**30**	21.0	Decane, 3,6-dimethyl-	C12H26	1.13
**31**	22.1	Heptadecane	C17H36	2.85
**32**	22.2	Pentadecane, 2,6,10,14-tetramethyl	C19H40	1.40
**33**	24.4	Octadecane	C18H38	2.22
**34**	24.6	Hexadecane, 2,6,10,14-tetramethyl-	C20H42	2.24
**35**	26.2	Heptadecane	C17H36	1.85
**36**	28.4	Octadecane	C18H38	2.04
**37**	30.2	Heneicosane	C21H44	1.08
**38**	31.8	Eicosane	C20H42	1.59
**39**	33.7	Heneicosane	C21H44	0.19

Table S4. Gas Chromatography Mass Spectrometry (GC-MS) of diesel

S.No	Retention Time	Compound Name	Chemical Formula	% Area
**1**	5.6	Benzene, 1-ethyl-3-methyl-	C9H12	4.10
**2**	6.1	Benzene, 1,2,3-trimethyl-	C9H12	5.47
**3**	6.6	Benzene, 1,2,3-trimethyl-	C9H12	2.13
**4**	6.7	Benzene, 2-ethyl-1,4-dimethyl-	C10H14	2.60
**5**	7.1	Benzene, 2-ethyl-1,4-dimethyl-	C10H14	2.20
**6**	7.3	Decane, 3-methyl-	C11H24	1.21
**7**	7.4	Benzene, 1-ethyl-2,4-dimethyl-	C10H14	1.35
**8**	7.6	Benzene, 1-ethyl-2,4-dimethyl-	C10H14	1.65
**9**	7.8	Undecane	C11H24	2.11
**10**	8.1	Naphthalene, decahydro-2-methyl	C11H20	1.48
**11**	8.9	Benzene, 1-methyl-2-(2-propenyl)-	C10H12	1.72
**12**	9.1	Undecane, 2-methyl-	C12H26	1.32
**13**	9.3	Undecane, 3-methyl-	C12H26	1.01
**14**	9.6	Napthalene, decahydro-1, 5-dimethyl	C12H22	1.41
**15**	10.2	Dodecane	C12H26	3.15
**16**	10.3	Undecane, 2,6-dimethyl-	C13H28	1.39
**17**	10.7	Naphthalene, 1,2,3,4-tetrahydro-1-methyl-	C11H14	1.19
**18**	11.0	Naphthalene, 1,2,3,4-tetrahydro-6-methyl-	C11H14	1.19
**19**	11.3	Benzene, (3-methyl-2-butenyl)-	C11H14	1.60
**20**	11.7	Naphthalene, 1,2,3,4-tetrahydro-6-methyl-	C11H14	1.12
**21**	12.4	Napthalene, 2-methyl-	C11H10	2.43
**22**	13.0	Naphthalene, 1,2,3,4-tetrahydro-1-4-dimethyl-	C12H16	1.82
**23**	13.6	Benzene, (3-methyl-2-butenyl)-	C11H14	0.90
**24**	14.0	Tridecane, 4-methyl-	C14H30	1.09
**25**	14.5	Dodecane, 2,6,10-trimethyl-	C15H32	2.30
**26**	14.9	Naphthalene, 1,2,3,4-tetrahydro-5,7-dimethyl	C10H12	1.25
**27**	15.2	Tetradecane	C14H30	4.80
**28**	15.6	Napthalene, 2,7-dimethyl-	C12H12	1.24
**29**	15.1	Napthalene, decahydro-1, 5-dimethyl	C12H22	1.43
**30**	16.6	Hexacosane	C16H34	1.65
**31**	16.8	Tetradecane, 3-methyl-	C15H32	1.19
**32**	17.6	Pentadecane	C15H32	4.24
**33**	18.3	Napthalene, 1,6,7-trimethyl-	C13H14	1.14
**34**	18.7	Napthalene, 1,4,6-trimethyl-	C13H14	1.02

**35**	19.0	Pentadecane, 2-methyl-	C16H34	1.43
**36**	20.0	Hexadecane	C16H34	3.71
**37**	21.0	Pentadecane, 2,6,10-trimethyl-	C18H38	1.54
**38**	21.5	Hexadecane, 3-methyl-	C17H36	0.90
**39**	22.3	Heptadecane	C17H36	4.15
**40**	22.4	Pentadecane, 2,6,10,14-tetramethyl-	C19H40	2.35
**41**	24.4	Octadecane	C18H38	3.31
**42**	24.5	Hexadecane, 2,6,10,14-tetramethyl-	C20H42	1.16
**43**	25.1	Octadecane	C18H38	0.92
**44**	26.4	Heptadecane	C17H36	2.84
**45**	28.3	Heptadecane	C17H36	2.88
**46**	30.2	Heneicosane	C21H44	2.67
**47**	31.9	Heptadecane, 3-methyl-	C18H38	2.65
**48**	33.6	Octadecane	C18H38	1.65
**49**	35.2	Tetracosane	C24H50	1.42
**50**	36.8	Tetracosane	C24H50	1.02
